# FvMAPK6‐Mediated FvMYB44s/FvSWEET1 Dual‐Layer Regulation Modulates Sugar Accumulation in Strawberry Fruit, With *FvSPS3* Enabling Quality–Yield Balance

**DOI:** 10.1111/pbi.70623

**Published:** 2026-03-13

**Authors:** Qianqian Feng, Lingzhi Wei, Ting Liu, Kexin Wang, Xiaojing Li, Chuang Liu, Ronghui Sun, Xia Li, Zhaonan Yin, Yanrong Wei, Huazhao Yuan, Qian Li, Bingbing Li

**Affiliations:** ^1^ Department of Pomology, College of Horticulture China Agricultural University Beijing China; ^2^ Institute of Forestry and Pomology Beijing Academy of Agriculture and Forestry Sciences Beijing China; ^3^ Key Laboratory of Plant Resources, Institute of Botany Chinese Academy of Sciences Beijing China; ^4^ Institute of Pomology Jiangsu Academy of Agricultural Sciences/Jiangsu Key Laboratory for Horticultural Crop Genetic Improvement Nanjing China

**Keywords:** FvMAPK6, FvMYB44.1/MYB44.2, FvSPS3, FvSWEET1, strawberry fruit, sugar accumulation

## Abstract

Sugar content is a key determinant of fruit quality, and sugars also act as signalling molecules that regulate ripening processes, including anthocyanin accumulation. However, the molecular mechanisms underlying sugar accumulation and sugar signal‐mediated ripening remain incompletely understood. In this study, we identify FvMAPK6 as an important phosphorylation hub that coordinates both sugar and anthocyanin accumulation in strawberry fruit. FvMAPK6 forms a phosphorylation cascade with FvMAPKK4, which directly phosphorylates the transcription factors FvMYB44.1 and FvMYB44.2. This phosphorylation reduces the stability and transcriptional activity of these proteins, attenuates their repression of downstream target genes such as *FvCHI*, *FvSPS3* and *FvSWEET1*, thereby coordinating anthocyanin and sugar accumulation. Furthermore, FvMAPK6 increases the protein abundance of the hexose transporter FvSWEET1 in strawberry fruits and alters its transport activity through phosphorylation. We demonstrate that sucrose treatment activates FvMAPK6, reinforcing its regulation of FvMYB44s and FvSWEET1 and thus amplifying sugar and anthocyanin accumulation. These findings establish FvMAPK6 as a key regulator that integrates both sugar accumulation and signalling at both transcriptional and post‐transcriptional levels. Although FvMAPK6 promotes sugar accumulation, it significantly reduces fruit yield and vegetative growth. To overcome this limitation, we screen for downstream targets of FvMAPK6 and identify *FvSPS3* as a promising breeding target: modulating *FvSPS3* improves fruit quality without compromising vegetative growth or yield. Collectively, our findings reveal novel regulatory pathways modulating sugar accumulation and signalling in strawberry while providing a valuable molecular target for the simultaneous improvement of fruit quality and agricultural productivity.

## 1 Introduction

Soluble sugars are fundamental determinants of fruit quality, serving not only as primary metabolites that influence flavour and sweetness, but also as signalling molecules that modulate broader ripening processes, including pigment accumulation (Rolland et al. 2002; Ruan 2014; Li and Sheen 2016; Durán‐Soria et al. 2020). However, despite their established roles, the molecular networks controlling sugar accumulation and the transduction of sugar signals during fruit ripening—particularly in non‐climacteric species—remain incompletely elucidated. A major challenge in fruit crop improvement lies in the frequent trade‐off between high sugar content (and associated quality traits) and yield (Zhang et al. 2024; Feldmann et al. 2024; Fan et al. 2024; Prohaska et al. 2024). Therefore, dissecting the regulatory mechanisms underlying sugar accumulation is crucial for devising strategies to simultaneously enhance fruit quality and agricultural productivity.

The woodland strawberry ( *Fragaria vesca* ) provides an excellent model system for studying fruit quality regulation in non‐climacteric fruits (Kang et al. 2013; Gaston et al. 2020). In strawberries, sucrose, glucose and fructose are the predominant sugars, with sucrose levels being a key contributor to SSC (soluble solids content) across different cultivars (Fait et al. 2008; Lee et al. 2018; Liu et al. 2020). Notably, sucrose itself functions as a signal to promote the accumulation of both sugars and anthocyanins (Jia et al. 2013; Duan et al. 2024), yet the specific signalling components and pathways mediating this sucrose‐driven coordination are poorly defined. Furthermore, the negative genetic correlation between SSC and yield observed in strawberry underscores the importance of understanding the genetic basis of this trade‐off for targeted breeding (Feldmann et al. 2024; Fan et al. 2024; Prohaska et al. 2024).

Fruit sugar content is determined by the interplay of metabolic enzymes and transport proteins, which mediate sugar biosynthesis, degradation and inter/intracellular transport. In strawberry, key candidate genes involved in these processes include sucrose phosphate synthase 3 (*FvSPS3*), sucrose synthases *1/2* (*FvSUS1/2*), and the sugar transporter Sugars Will Eventually be Exported Transporter 1 (FvSWEET1), whose expression correlates with sugar accumulation during ripening (Vallarino et al. 2015; Jia et al. 2017; Lee et al. 2018; Liu et al. 2019). Nevertheless, definitive genetic evidence for their roles in ripening and their potential for breeding applications remains limited.

At the transcriptional level, MYB transcription factors are important regulators of sugar metabolism and anthocyanin biosynthesis (Liu et al. 2023, 2025; Li et al. 2021; Gao, Yang, et al. 2023; Wang et al. 2024). In cultivated strawberry (*F. × ananassa*), FaMYB44.2 has been shown to repress sugar accumulation by directly targeting genes such as *FaSPS3* and *FaSUS1*, while also indirectly modulating anthocyanin pathways by competing with the activator FaMYB10 (Wei et al. 2018). Homologues of MYB44 influence carbohydrate metabolism in diverse plant species including wheat ( *Triticum aestivum* ), rice (*Oryza sativa*), melon ( *Cucumis melo* ) and grape ( *Vitis vinifera* ) (Li et al. 2021; Gao, Yang, et al. 2023; Wang et al. 2024; Liu et al. 2025), highlighting a conserved role in carbon partitioning. However, the upstream regulators that control MYB44 activity in carbon partitioning, particularly in response to sugar signals, remain largely unknown.

Post‐translational modifications, particularly phosphorylation, represent a critical layer in the regulation of sugar metabolism. Evidence from diverse fruit crops underscores this point: the malectin domain‐containing receptor‐like kinase FaMRLK47 and the SNF1 (sucrose non‐fermenting 1)‐related protein kinases 1 α‐subunit (FvSnRK1α) were identified as regulators of sugar accumulation in strawberry fruits (Jia et al. 2017; Luo et al. 2020); The SNF1‐related protein kinase 2 (SnRK2) family members ClSnRK2.3 and MdSnRK2.3 can have both positive and negative effects on sugar accumulation in watermelon ( *Citrullus lanatus* ) and apple ( *Malus domestica* ) fruits, respectively, via phosphorylation of their downstream targets (Wang et al. 2023; Zhu et al. 2023). Recently, *Solanum lycopersicum* Calcium‐Dependent Protein Kinase 26/27 (SlCDPK26/27) have been identified as the ‘sugar brake’, which involves phosphorylating SlSUS3 to regulate the accumulation of hexose in tomato fruit (Zhang et al. 2024). A key finding from *Arabidopsis* further solidified this concept by demonstrating that AtSnRK2s regulate sugar allocation by phosphorylating the sucrose transporters AtSWEET11 and AtSWEET12 (Chen et al. 2022). This finding directly highlights the critical role of phosphorylation in modulating SWEET protein function. However, given that SWEET proteins are heptahelical transmembrane transporters—a class notoriously difficult to study—our understanding of their regulatory mechanisms remains limited (Ji et al. 2022). Consequently, it is unclear whether this phosphorylation‐dependent regulatory mechanism applies to other SWEET family members involved in transporting diverse sugars. Moreover, the upstream signals triggering such phosphorylation events remain to be elucidated; specifically, it is essential to determine whether these signals can be activated by certain sugars acting as signalling molecules, independent of their function as transport substrates. These findings highlight the essential role of phosphorylation in regulating fruit sugar accumulation. While kinases like SnRKs and CDPKs are established regulators, the potential roles of other kinase families in fruit sugar accumulation remain largely unexplored, highlighting a significant gap in our knowledge.

Mitogen‐activated protein kinases (MAPKs), which are typically activated by upstream MAPKKs/MAPKKK to form phosphorylation cascades, are key signalling modules that translate various stimuli into cellular responses (Zhang et al. 2018; Zhang and Zhang 2022). Although MAPK pathways are well‐studied in model plants such as *Arabidopsis*, their specific contribution to sugar accumulation remains largely unknown. For instance, while AtMAPK3 and AtMPK6 are involved in glucose signalling during seed germination (Bhagat et al. 2022), no evidence establishes a direct link to controlling actual sugar levels in vegetative or reproductive tissues. Thus, it is unknown whether MAPK cascades contribute to fruit sugar accumulation or how they might integrate sugar signalling with ripening processes. Furthermore, the potential for a single MAPK to coordinate multiple processes by phosphorylating distinct substrates, such as transcription factors and metabolite transporters, which represents a ‘phosphorylation hub’ strategy, remains an intriguing but untested hypothesis in the context of fruit quality regulation.

To bridge these knowledge gaps, we identified FvMAPK6 as a sucrose‐responsive kinase interacting with FvMYB44s. We demonstrate that FvMAPK6 coordinates sugar and anthocyanin accumulation in strawberry fruit via a dual mechanism: it directly phosphorylates and modulates the stability and activity of transcription factors FvMYB44.1 and FvMYB44.2, and simultaneously phosphorylates and enhances the activity of the sugar transporter FvSWEET1. Sucrose treatment amplifies the regulation of FvMAPK6 on FvMYB44 and FvSWEET1 mediated sugar accumulation, implying that FvMAPK6 acts as an important phosphorylation hub in a sucrose‐mediated positive feedback loop. While enhancing FvMAPK6 activity improved fruit quality, it also incurred a yield penalty. To overcome this trade‐off, we screened downstream targets and identified *FvSPS3* as a key effector; modulating *FvSPS3* expression successfully enhanced sugar content without compromising plant growth or yield. Our work thus unveils novel regulatory pathways for sugar accumulation and provides a viable breeding strategy for quality improvement in strawberry.

## 2 Results

### 2.1 FvMAPK6 Regulates the Coordinated Accumulation of Sugar and Anthocyanin in Strawberry Fruits

We previously revealed that the transcription factor FvMYB44.2 is a key regulator of sucrose accumulation (Wei et al. 2018). To identify proteins that directly interact with FvMYB44.2 and are potentially involved in sugar accumulation, we conducted a liquid chromatography–mass spectrometry (LC–MS) analysis to screen for its binding partners, leading to the identification of FvMAPK6 (Figure S1; Table S1).

We then generated stable overexpression (OE) transgenic lines and genome‐edited (cr) mutant lines via clustered regularly interspaced short palindromic repeats (CRISPR)/CRISPR‐associated nuclease 9 (Cas9)‐mediated gene editing in a diploid strawberry background ( *F. vesca* cv. Ruegen) to examine the roles of *FvMAPK6* in fruit ripening. While both the gene expression and kinase activity of FvMAPK6 were significantly upregulated in the overexpression lines (Figure S2A,B), FvMAPK6 activity notably decreased in the biallelic mutant lines (−3 bp/−5 bp) and were completely abolished in the homozygous mutant lines (−5 bp/−5 bp) (Figure S2C,D). However, the homozygous mutant lines were sterile (Figure S2E). Therefore, we used fruits from biallelic plants (*Fvmapk6*‐cr‐2) and its T2 biallelic generation (*Fvmapk6*‐cr‐2‐1) to analyse the function of *FvMAPK6* (Figure S2F–I).

**FIGURE 1:**
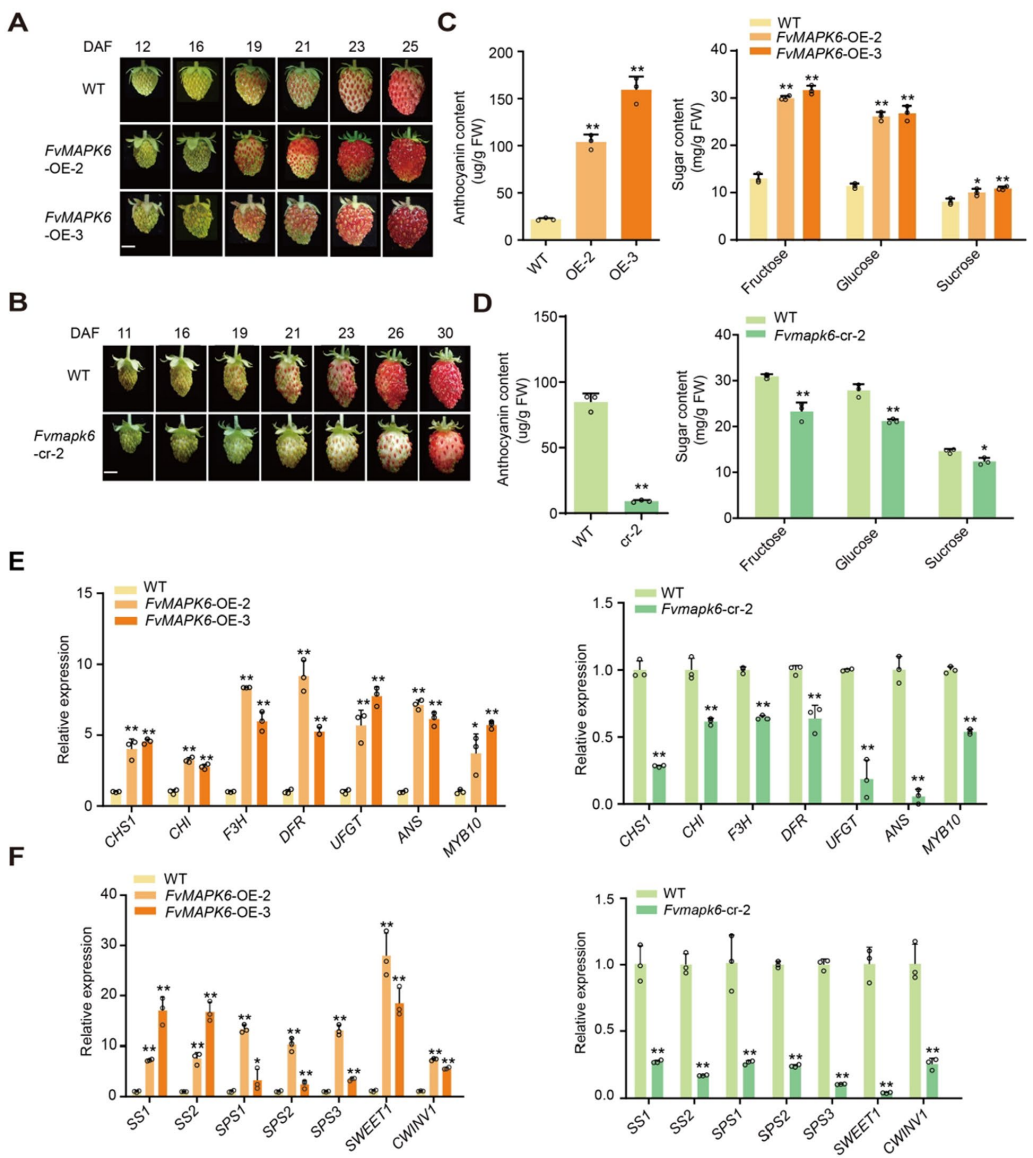
FvMAPK6 regulates the coordinated accumulation of anthocyanins and sugars in strawberry fruits. (A, B) Phenotypes of wild‐type (WT), *FvMAPK6*‐OE‐2, OE‐3, and *Fvmapk6*‐cr‐2 fruits at different days after fruit setting (DAF). Scale bars: 0.5 cm. (C, D) Anthocyanin and sugar contents of WT and *FvMAPK6*‐OE‐2, OE‐3 at 25 DAF (C) or WT and *Fvmapk6*‐cr‐2 fruits at 26 DAF (D). (E, F) Expression levels of genes related to anthocyanin biosynthesis, sugar metabolism, and transport, as measured by RT‐qPCR using the fruits described in (C) and (D). Values are means ± standard deviation (± SD) from three independent biological replicates; each replicate consisted of 15 fruits. Significance was assessed using Student's *t*‐test (two‐sided; *p < 0.05, **p < 0.01).

Compared to the wild type (WT), *FvMAPK6*‐OE fruits showed accelerated anthocyanin accumulation, while *Fvmapk6*‐cr‐2 and *Fvmapk6‐cr‐2‐1* fruits showed delayed anthocyanin accumulation (Figure 1A–D; Figure S2F). The accumulation patterns of sugars, including glucose, fructose and sucrose, were consistent with those of anthocyanins in *FvMAPK*6‐OE and *Fvmapk6*‐cr fruits (Figure 1A–D; Figure S2F–H). RT‐qPCR and RNA‐seq analysis of genes involved in anthocyanin and sugar accumulation revealed that FvMAPK6 regulates the expression of key structural genes, including *FvCHS1*, *FvCHI*, *FvSS1*, *FvSS2*, *FvSPS3*, *FvSWEET1* and *FvCWINV1* (Figure 1E,F; Figure S2I; Table S2). However, the changes in the expression of sugar‐related genes were more pronounced than those for genes related to anthocyanin biosynthesis (Figure 1E,F), suggesting that *FvMAPK6* might play a more important role in sugar accumulation. Additionally, *FvMAPK6* regulated the expression of transcription factor genes associated with strawberry fruit ripening, such as *FvMYB10*, *FvMYB44.2*, *FvWRKY50* and *FvASR*, among others (Figure 1E; Table S2) (Liu et al. 2015; Wei et al. 2018; Chen et al. 2023). However, the expression pattern of *FvMYB44.2* we observed here contradicts its reported function in regulating sucrose accumulation in strawberry fruits (Wei et al. 2018). Therefore, FvMAPK6 might regulate the activities of some transcription factors at the post‐transcriptional rather than the transcriptional level.

### 2.2 FvMAPK6 Phosphorylates FvMYB44.1 and FvMYB44.2 to Modulate Their Degradation and Transcriptional Activity

**FIGURE 2:**
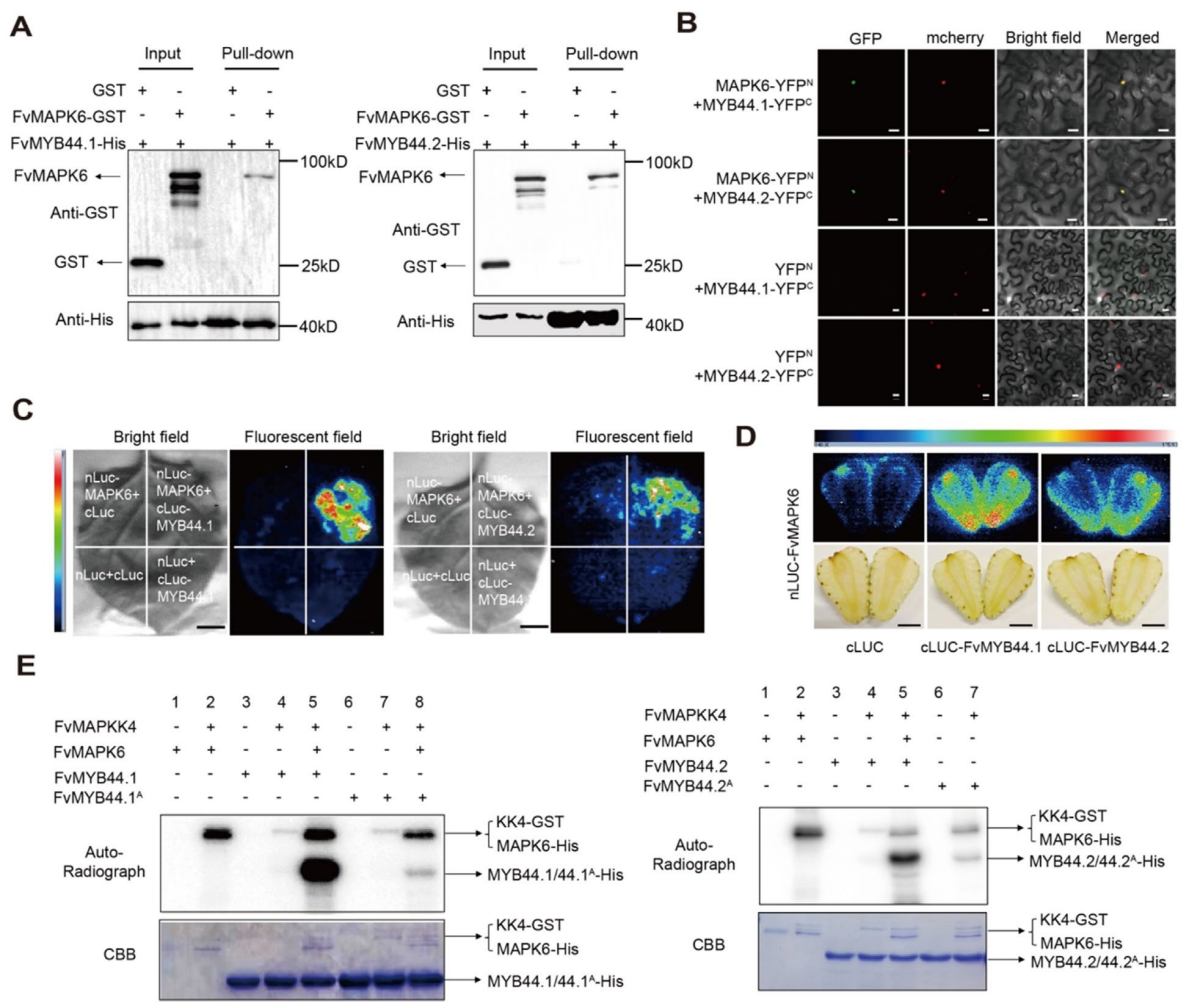
FvMAPK6 directly interacts with and phosphorylates FvMYB44.1 and FvMYB44.2. (A‐D) Analysis of the interactions between FvMAPK6 and FvMYB44.1/FvMYB44.2 using a pull‐down assay (A), BiFC assay (B), and LCI assay in *N*. *benthamiana* leaves (C) and strawberry fruits (D). Scale bars, 50 μm in (B), 1 cm in (C, D). (E) In vitro phosphorylation assays revealing that the FvMKK4–FvMAPK6 module phosphorylates FvMYB44.1 at Ser‐62 and FvMYB44.2 at Ser‐226.

As FvMYB44.2 has been identified as the binding partner of FvMAPK6 in strawberry fruit, we further validated their direct interaction both in vitro and in vivo via pull‐down, BiFC, and LCI assays (Figure 2A–D). In addition to modulating *FvMYB44.2* expression, FvMAPK6 also affected the expression of *FvMYB44.1* (Table S2); we further examined the interaction between FvMAPK6 and FvMYB44.1 using the same experimental approaches, which confirmed their physical association (Figure 2A–D).

To determine whether FvMYB44.1 and FvMYB44.2 are phosphorylated substrates of FvMAPK6, we conducted in vitro phosphorylation assays. However, since MAPKs typically require activation by upstream MAPKKs to exhibit kinase activity and the recombinant purified FvMAPK6 lacks autophosphorylation capability, we screened for its interacting upstream FvMAPKKs. Through yeast two‐hybrid and IP‐MS analysis using *FvMAPK6*‐OE fruits, we identified FvMAPKK4 as an interacting partner of FvMAPK6 (Figure S3A,B; Table S3). Furthermore, FvMAPKK4 exhibited significantly higher transcript levels than those of other FvMAPKK family members in strawberry fruit (Figure S3C). These findings led to the identification of FvMAPKK4 as the upstream kinase of FvMAPK6. Further analysis demonstrated that FvMAPKK4 indeed activated FvMAPK6, and the activated FvMAPK6 subsequently phosphorylated FvMYB44.1 at Ser‐62 and FvMYB44.2 at Ser‐226, as determined by LC–MS and in vitro kinase assays (Figure 2E; Figure S4). The phosphorylation of their phosphor‐dead variants, FvMYB44.1^A^ and FvMYB44.2^A^, was almost completely abolished compared to FvMYB44.1 and FvMYB44.2 (Figure 2E).

To investigate the function of FvMYB44.1, we transiently overexpressed (*FvMYB44.1*‐OE) or silenced (via RNA interference [RNAi]; *FvMYB44.1*‐RNAi) *FvMYB44.1* in strawberry fruits (Figure S5A–C). Whereas *FvMYB44.2* primarily regulated sucrose accumulation in strawberry fruits (Wei et al. 2018), *FvMYB44.1* negatively regulated anthocyanin, sucrose, fructose and glucose accumulation by affecting the expression of genes involved in anthocyanin biosynthesis, sugar metabolism, and sugar transport (Figure S5D–F). FvMYB44.1 directly bound to the promoters of key genes such as *FvCHI*, *FvSPS3*, *FvSUS2* and *FvSWEET1*, as observed with EMSA using recombinant purified FvMYB44.1 (Figure S5G). We further demonstrated that FvMYB44.1 represses the expression of its downstream genes involved in sugar and anthocyanin accumulation by evaluating its effect on GUS activity driven by the promoters of *FvCHI*, *FvSPS3*, *FvSUS2* and *FvSWEET1* in strawberry fruit. (Figure S5H).

**FIGURE 3:**
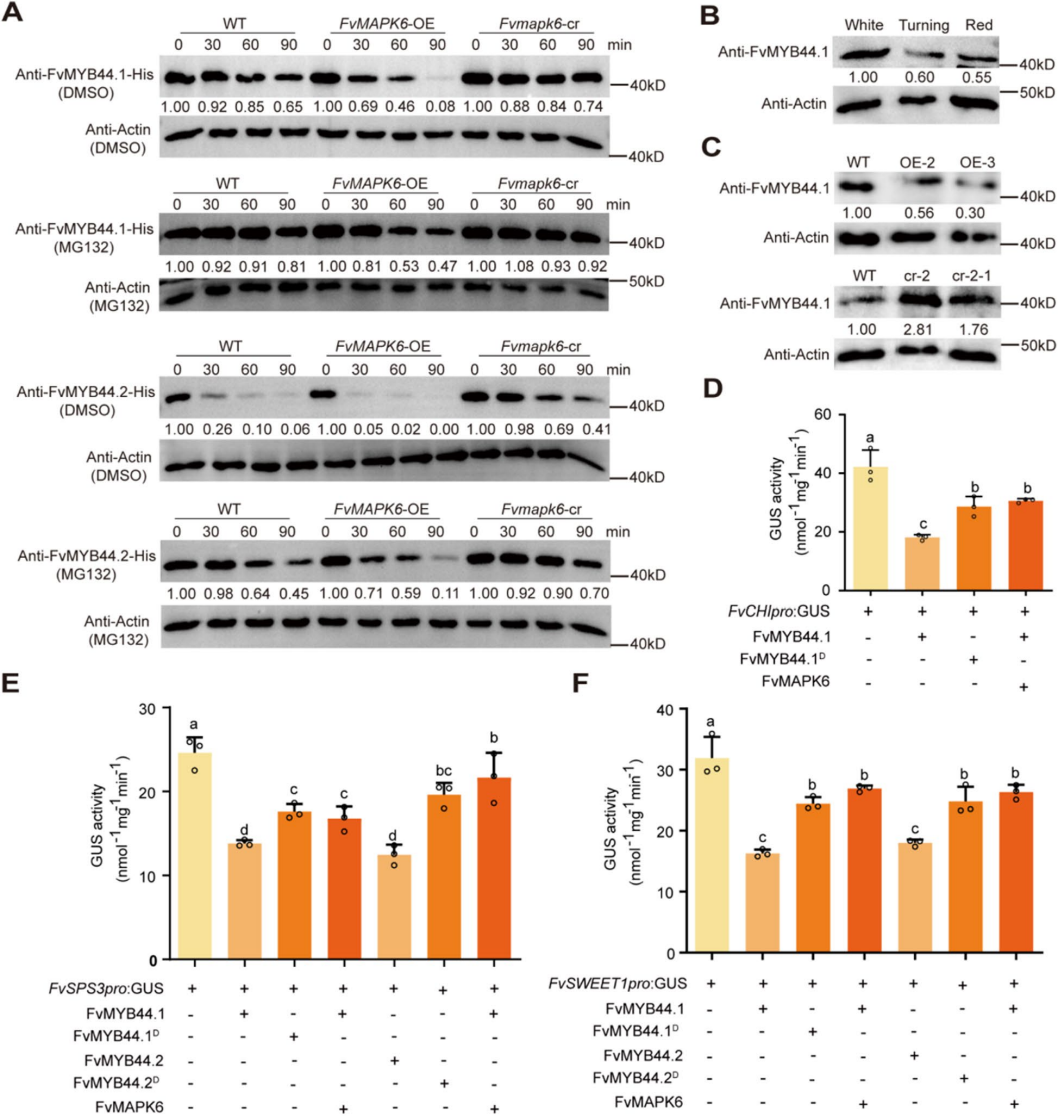
FvMAPK6 leads to the protein degradation of transcription factor FvMYB44.1/FvMYB44.2 and alleviates their repression of downstream genes. (A) Cell‐free degradation assay demonstrating that FvMAPK6 promotes the degradation of recombinant purified FvMYB44.1/MYB44.2‐His proteins. Recombinant FvMYB44.1/MYB44.2‐His proteins were added to total protein extracts from WT, *FvMAPK6*‐OE or *Fvmapk6*‐cr fruits and incubated with 50 μM MG132 or DMSO (control) at 37°C, and FvMYB44.1/MYB44.2‐His proteins were detected using anti‐His antibody. (B, C) Immunoblot assay to detect FvMYB44.1 abundance in WT fruits at different developmental stages (B), as well as red fruits of WT, *FvMAPK6*‐OE and *Fvmapk6*‐cr (C). FvMYB44.1 was recognised by the anti‐FvMYB44.1 antibody. (D–F) Analysis of the effect of phosphorylation on the transcriptional activity of FvMYB44s using effector constructs expressing wild‐type (*FvMYB44.1*, *FvMYB44.2*) and the phospho‐mimetic mutant (*FvMYB44.1* ^*D*^, *FvMYB44.2* ^*D*^) co‐infiltrated ‘Benihoppe’ strawberry fruits with reporter constructs such as *GUS* driven by the promoter of a flavonoid biosynthesis gene (*FvCHIpro*:*GUS*), sucrose phosphate synthase gene (*FvSPS3pro*:*GUS*) or sugar transporter gene (*FvSWEET1pro*:*GUS*). ‘+’ and ‘−’ represent the presence or absence of the corresponding plasmid in Agrobacterium, respectively. Values are means ± SD. (*n* = 3 independent biological replicates; each replicate contained 10 fruits). Different lowercase letters in D–F indicate significant differences, as assessed by one‐way ANOVA (with Tukey's test) (*p* < 0.05).

To further elucidate the biological significance of FvMAPK6‐mediated phosphorylation of FvMYB44, we investigated the effects of FvMAPK6 on the protein levels and transcriptional activity of FvMYB44.1 and FvMYB44.2. Compared to incubation with WT fruit extracts, incubation with *FvMAPK6*‐OE fruit extracts significantly promoted the degradation of both FvMYB44.1 and FvMYB44.2. Furthermore, the proteasome inhibitor MG132 significantly reduced FvMAPK6‐mediated degradation of FvMYB44.1 and FvMYB44.2, suggesting that FvMAPK6 facilitates the degradation of these proteins via the 26S‐proteasome pathway (Figure 3A). We next developed an endogenous antibody against FvMYB44.1 to monitor its protein levels in strawberry fruits. The abundance of FvMYB44.1 was significantly reduced at the turning and red fruit stages compared with the white fruit stage, suggesting a decline in protein accumulation during fruit ripening (Figure 3B). We further analysed FvMYB44.1 protein levels in *FvMAPK6* transgenic red fruits (Figure 3C). The results revealed a marked decrease in *FvMAPK6*‐OE fruits and a clear increase in *Fvmapk6*‐cr fruits, consistent with results from the cell‐free protein degradation assay.

To investigate the effect of FvMAPK6‐mediated phosphorylation on the transcriptional activity of FvMYB44.1 and FvMYB44.2, we employed a transcriptional reporter assay in strawberry fruits. In this assay, the β‐glucuronidase (GUS) reporter gene was driven by the promoters of the downstream structural genes *FvCHI*, *FvSPS3* and *FvSWEET1*. We observed that transient overexpression of *FvMYB44s* significantly downregulated the expression of these genes (Figure 3D–F). However, this downregulation was counteracted either by co‐overexpressing *FvMAPK6* with *FvMYB44.1/FvMYB44.2*, or by overexpressing the phosphomimetic variants *FvMYB44.1* ^*D*^ */MYB44.2* ^*D*^ alone (Figure 3D–F).

These findings demonstrate that the FvMAPKK4–FvMAPK6 cascade negatively regulates both the degradation and transcriptional activity of FvMYB44.1 and FvMYB44.2 in strawberry fruits via phosphorylation at residues Ser‐62 and Ser‐226, respectively.

### 2.3 FvMAPK6 Phosphorylates the Hexose Transporter FvSWEET1 to Regulate Its Function

**FIGURE 4:**
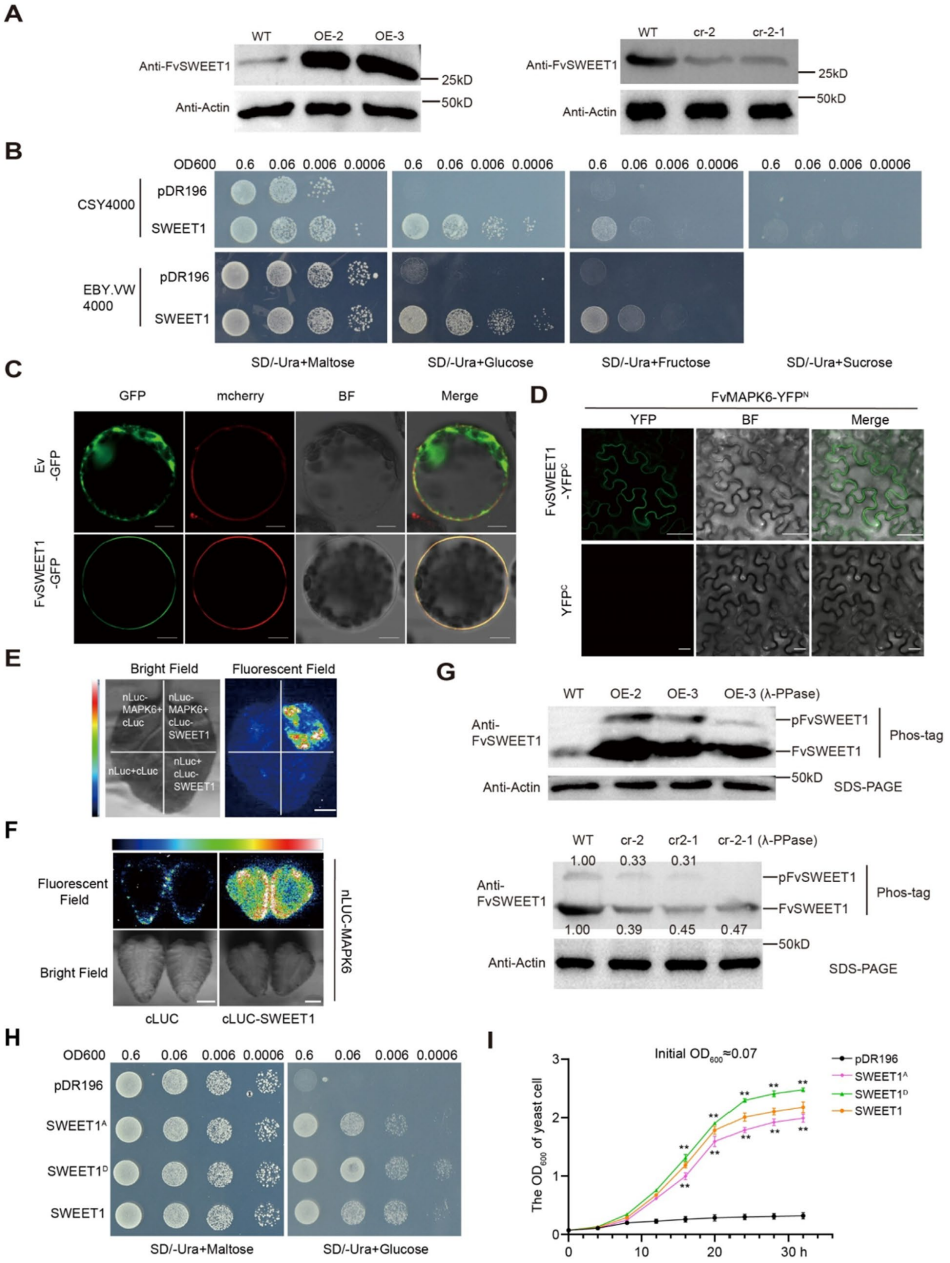
FvMAPK6 increases the protein abundance of the hexose transporter FvSWEET1 in strawberry fruits and alters its transport activity through phosphorylation. (A) Immunoblotting to detect FvSWEET1 levels in WT, *FvMAPK6*‐OE, and *Fvmapk6‐*cr red fruits. (B) Growth complementation assays of the hexose and sucrose uptake‐deficient yeast strain CSY4000 or hexose uptake‐deficient yeast strain EBY.VW4000 heterologously expressing *FvSWEET1*, respectively. Yeast growth was assessed on synthetic defined medium lacking uracil (SD/−Ura) supplemented with different carbon sources: 2% (w/v) maltose, 2% (w/v) fructose, 2% (w/v) glucose or 2% (w/v) sucrose; yeast transformed with the empty vector (pDR196) was used as a negative control. (C) FvSWEET1 localises to the plasma membrane, as observed in *N*. *benthamiana* leaf protoplasts infiltrated with *35S*:*FvSWEET1‐GFP*. An mCherry‐labelled plasma membrane marker (AtCBL1) was co‐expressed to visualise the plasma membrane. Scale bars, 10 μm. (D) BiFC assay demonstrating the interaction between FvMAPK6 and FvSWEET1 at the plasma membrane of *N. benthamiana* leaf cells. Scale bars, 25 μm. (E, F) LCI assay revealing the interaction between FvMAPK6 and FvSWEET1 in *N*. *benthamiana* leaves (E) or strawberry fruits (F). Scale bars, 1 cm. (G) The phosphorylation status of FvSWEET1 in WT, *FvMAPK6*‐OE, and *Fvmapk6*‐cr red strawberry fruits was assessed using a phos‐tag assay. Phosphorylated and non‐phosphorylated FvSWEET1 (p‐FvSWEET1 and FvSWEET1) were detected with anti‐FvSWEET1 antibody. *β*‐actin levels were used as an input control for SDS‐PAGE; λ‐PPase, λ‐phosphatase. (H) Serial dilutions of EBY.VW4000 cells transformed with SWEET1 and its Ser223 mutant (pDR196‐SWEET1^A/D^) in pDR196 were cultured on SC/‐Ura medium containing 2% maltose or glucose. Yeast cells carrying the empty pDR196 vector served as control. Spot dilutions were performed from left to right in 10‐fold increments, starting with an initial OD600 of 0.6. (I) EBY.VW4000 yeast cells expressing pDR196‐SWEET1, its Ser223 mutant (pDR196‐SWEET1^A/D^) or the empty vector pDR196 (control), were diluted in liquid SC/‐Ura medium supplemented with 2% glucose and cultured at 30°C in a shaking incubator. Cell density was measured at 4‐h intervals over a 32 h period following treatment. Values are means ± SD. from three independent biological replicates. Significance was assessed using Student's *t*‐test (two‐sided; *p < 0.05, **p < 0.01).

In addition to regulating the expression of downstream structural genes by phosphorylating transcription factors, protein kinases can also directly phosphorylate functional proteins to exert their biological functions. However, compared with transcription factors, the identification of this type of substrate is relatively scarce at present in fruits. To gain a more comprehensive understanding of the mechanism underlying *FvMAPK6* function, we conducted a phosphoproteomic analysis using *FvMAPK6* transgenic fruits. FvMAPK6 significantly modulated the phosphorylation levels of proteins related to sugar transport, metabolism and signal transduction (Figure S6). Notably, the phosphorylation level of FvSWEET1, the highest‐expressed member in the *SWEET* family whose expression is significantly regulated by *FvMAPK6*, was significantly altered (Figure S7; Figure S8A,B). We next developed an endogenous antibody against FvSWEET1. Protein level analysis revealed that FvSWEET1 accumulation was higher in *FvMAPK6*‐OE fruits but lower in *Fvmapk6*‐cr fruits compared to WT fruits (Figure 4A). This result is consistent with our finding that FvMAPK6 regulates FvMYB44.1 to attenuate its repression of the direct downstream gene *FvSWEET1* (Figure S5).

Phylogenetic tree analysis showed that *FvSWEET1* belonged to *SWEET* subfamily I and might function as a hexose transporter (Figure S8C). To assess its transport activity, *FvSWEET1* was heterologously expressed in hexose/sugar uptake‐deficient yeast strains and subjected to growth complementation assays. While FvSWEET1 transported glucose and fructose into yeast cells, it failed to transport sucrose, indicating that it functions as a hexose transporter (Figure 4B). Furthermore, we detected FvSWEET1 localisation at the plasma membrane in *N. benthamiana* protoplasts transformed with a FvSWEET1‐GFP construct (Figure 4C), and observed an interaction between FvMAPK6 and FvSWEET1 in both *N. benthamiana* leaves and strawberry fruits (Figure 4D–F).

Next, we investigated the phosphorylation‐mediated regulatory mechanism of FvMAPK6 on FvSWEET1. A phos‐tag assay revealed a significant alternation in FvSWEET1 phosphorylation in *FvMAPK6*‐OE fruit or *Fvmapk6*‐cr fruit relative to the WT that was greatly decreased by treating the protein extracts with λ‐phosphatase (Figure 4G). Phosphoproteomics assays identified phosphorylation at Ser residue 223 within the C terminus of FvSWEET1 (Figure S7). To investigate the functional impact of this phosphorylation, we generated phospho‐mimic and phosphor‐dead mutants of FvSWEET1 by substituting serine 223 with aspartic acid (D) or alanine (A), respectively. As shown in Figure 4H,I, compared to wild‐type FvSWEET1, the FvSWEET1^D^ mutant exhibited enhanced glucose transporter activity, while the FvSWEET1^A^ mutant showed reduced transporter activity. These data demonstrated that FvMAPK6‐mediated phosphorylation is critical for maintaining its transport activity.

### 2.4 Sucrose Signalling Activates the FvMAPK6–FvMYB44s/FvSWEET1 Dual Regulatory Module to Modulate Sugar Accumulation in Strawberry Fruits

**FIGURE 5:**
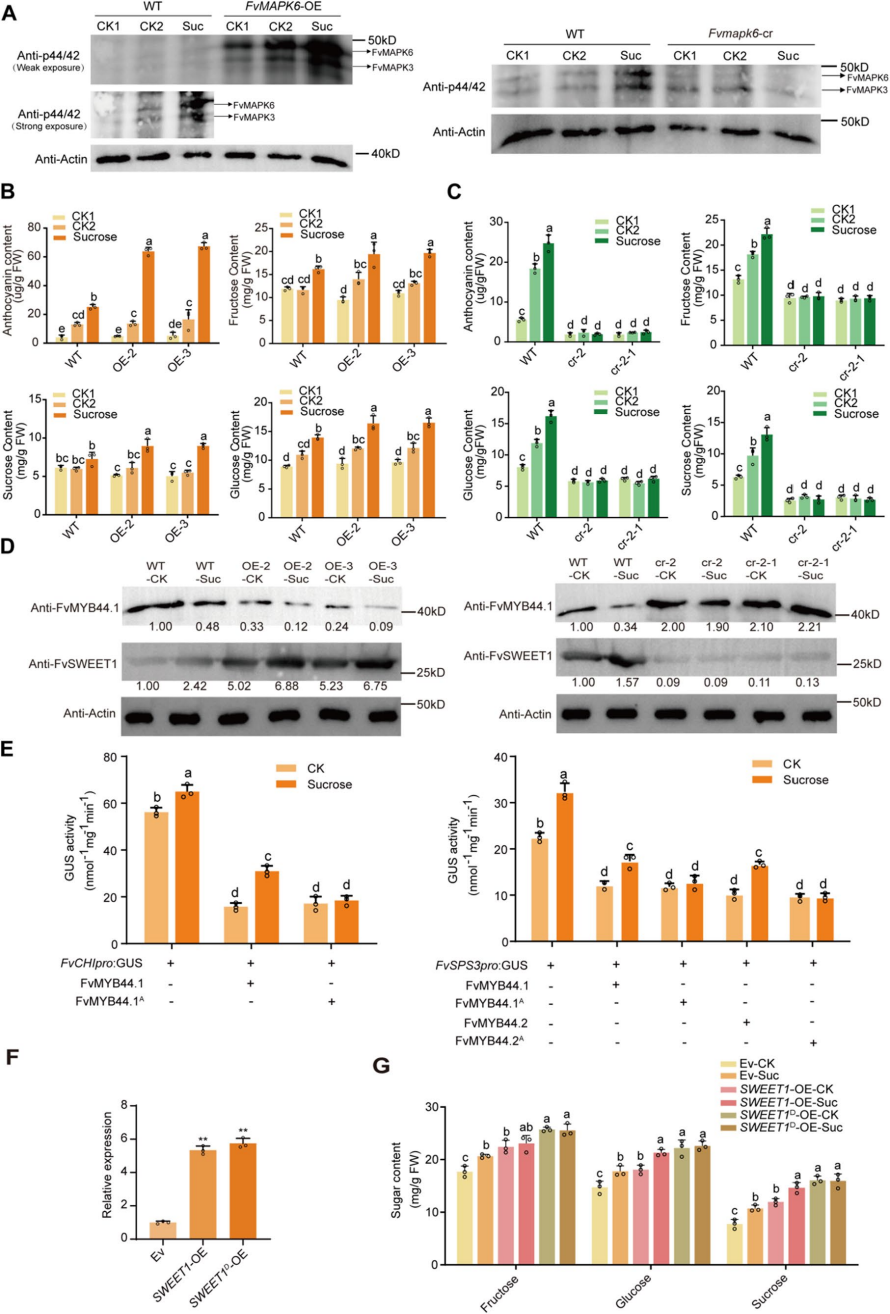
Sucrose signals activate FvMAPK6 to accelerate sugar and anthocyanin accumulation in strawberry fruits. (A) Immunoblot showing that the phosphorylation of FvMAPK6 is significantly activated by sucrose in white fruits of *FvMAPK6*‐OE. CK1, white fruits treated with water for 2 h; CK2, white fruits treated with 50 mM mannitol for 2 h; Suc, white fruit treated with 50 mM sucrose for 2 h. (B, C) Anthocyanin (B) and sugar (C) content in treated fruits from WT, *FvMAPK6*‐OE, and *Fvmapk6‐*cr. All fruits were subjected to treatment during the white fruit stage, with samples collected 2 days after treatment. CK1, white fruit treated with water; CK2, white fruit treated with 50 mM mannitol; Suc, white fruit treated with 50 mM sucrose. (D) The protein expression levels of FvMYB44.1 and FvSWEET1 were analysed by Western blotting in treated fruits as described in (B). CK, white fruit treated with water; Suc, white fruit treated with 50 mM sucrose. (E) The sucrose‐mediated activation of *FvCHI* and *FvSPS3* expression is hindered by the phosphor‐dead variants of FvMYB44.1. ‘Benihoppe’ fruits were co‐infiltrated with an effector construct (*FvMYB44.1*, *MYB44.1* ^*A*^, *MYB44.2* or *MYB44.2* ^*A*^) and *FvCHIpro*:*GUS* or *FvSPS3pro*:*GUS*, incubated for 24 h, and treated with 50 mM sucrose; water served as the control. The fruits were harvested after 24 h of sucrose treatment for the GUS activity assay. (F, G) Transient expression assay revealed that sucrose treatment activated *FvSWEET1*. ‘Benihoppe’ fruits were infiltrated with agrobacterium harbouring an empty vector (Ev), transiently overexpressing *FvSWEET1* and its phosphor‐mimic vector (*FvSWEET1*‐OE, *FvSWEET1* ^*D*^‐OE). After a 24‐h incubation period, fruits were randomly selected for determining the expression level of target genes using RT‐qPCR (F), and the remaining fruits were further treated with 50 mM sucrose or CK buffer (0 mM sucrose) for 24 h and then harvested for sugar content determination (G). Values are means ± SD. (*n* = 3 independent biological replicates; each replicate contained 15 fruits). Significance in *F* was determined using Student's *t*‐test (two‐sided; *p < 0.05, **p < 0.01). Different lowercase letters in B, C, E, G indicate significant differences, as assessed by one‐way ANOVA (with Tukey's test) (*p* < 0.05).

Considering the well‐documented role of 50 mM sucrose in mimicking sucrose signalling and promoting strawberry fruit ripening, including sugar and anthocyanin accumulation (Jia et al. 2013), we investigated whether FvMAPK6 is activated to participate in the sucrose signal‐mediated ripening process. A 50 mM mannitol treatment was used as an osmotic control. Our results showed that sucrose treatment significantly activated FvMAPK6 in WT fruits. This activation was further enhanced in *FvMAPK6*‐OE fruits but largely abolished in *Fvmapk6*‐cr‐2 fruits (Figure 5A). Collectively, these data demonstrate that FvMAPK6 is a key component of the sucrose signalling pathway in strawberry fruit cells.

To further elucidate the role of FvMAPK6 in sucrose‐mediated ripening, we investigated the effects of sucrose treatment on anthocyanin and sugar accumulation in *FvMAPK6*‐OE and *Fvmapk6‐cr* fruits. Compared to WT, sucrose treatment led to a greater enhancement of anthocyanin and sugar accumulation in *FvMAPK6*‐OE fruits, while this enhancement was notably delayed in *Fvmapk6‐cr* fruits (Figure 5B,C). We further examined the protein levels of the downstream phosphorylation targets of FvMAPK6, FvMYB44.1 and FvSWEET1, under sucrose treatment conditions. The results showed that sucrose treatment significantly altered the protein levels of FvMYB44.1 and FvSWEET1 in a FvMAPK6‐dependent manner (Figure 5D). These results suggest that the FvMAPK6–FvMYB44.1/FvSWEET1 module is a key component enabling strawberry fruit cells to respond to sucrose signalling.

We then investigated how FvMAPK6 modulates downstream gene expression in response to sucrose signalling. RT‐qPCR analysis revealed that the expression of key genes involved in anthocyanin biosynthesis (e.g., *FvCHI*) and sugar metabolism/transport (e.g., *FvSPS3* and *FvSWEET1*) was affected by FvMAPK6 in response to sucrose signalling (Figure S9). To test whether FvMAPK6 modulates this response through the phosphorylation of FvMYB44 transcription factors, we employed a transcriptional reporter assay. We generated strawberry fruits overexpressing *FvMYB44.1*, *FvMYB44.2*, or their respective phospho‐dead variants (FvMYB44.1^A^, FvMYB44.2^A^), using the promoters of the sucrose‐responsive genes *FvSPS3* and *FvCHI* to drive GUS expression. Sucrose treatment upregulated the activity of both promoters. However, this upregulation was attenuated by the overexpression of either wild‐type FvMYB44.1 or FvMYB44.1^A^ (Figure 5E). Notably, the phospho‐dead variant FvMYB44.1^A^ completely suppressed the sucrose‐induced activation, contrasting with the partial effect of wild‐type FvMYB44.1. A similar regulatory pattern was observed for FvMYB44.2 and FvMYB44.2^A^ on the *FvSPS3* promoter (Figure 5E). These results indicate that FvMAPK6 fine‐tunes the sucrose response through phosphorylation of FvMYB44s, which subsequently modulate the expression of genes like *FvCHI* and *FvSPS3*.

In parallel to the transcriptional regulatory pathway, we investigated whether FvMAPK6 mediates sucrose signalling by directly regulating the hexose transporter FvSWEET1. A critical question was whether sucrose acts as a signalling molecule or indirectly as a metabolic substrate. To test this, we analysed the effect of sucrose treatment on strawberry fruits overexpressing either wild‐type *FvSWEET1* or a phospho‐mimetic mutant (*FvSWEET1* ^*D*^). Both constructs significantly increased the basal levels of hexose and sucrose in strawberry fruits even without sucrose treatment, confirming that enhanced FvSWEET1 activity promotes sugar accumulation (Figure 5F,G). Notably, sucrose treatment further enhanced sugar accumulation only in fruits expressing wild‐type *FvSWEET1*, with no additive effect observed in those expressing the phospho‐mimetic *FvSWEET1* ^*D*^ mutant (Figure 5G). This absence of an additive effect in the constitutively active mutant provides key evidence that sucrose acts primarily as a signalling molecule to enhance FvSWEET1‐mediated hexose transport in strawberry, rather than functioning merely as a metabolic precursor. The results establish that FvMAPK6‐mediated phosphorylation is essential for the sucrose‐signalling‐dependent activation of FvSWEET1.

In summary, these findings demonstrate that FvMAPK6 acts as a central hub in sucrose signalling, coordinating sugar and anthocyanin accumulation in strawberry fruits by phosphorylating distinct downstream targets: the transcriptional regulators FvMYB44s to modulate gene expression networks, and the transporter FvSWEET1 to influence hexose uptake.

### 2.5 *FvSPS3* Increased Sugar Content in Strawberry Fruit Without Causing Adverse Growth‐Related or Yield Effects

**FIGURE 6:**
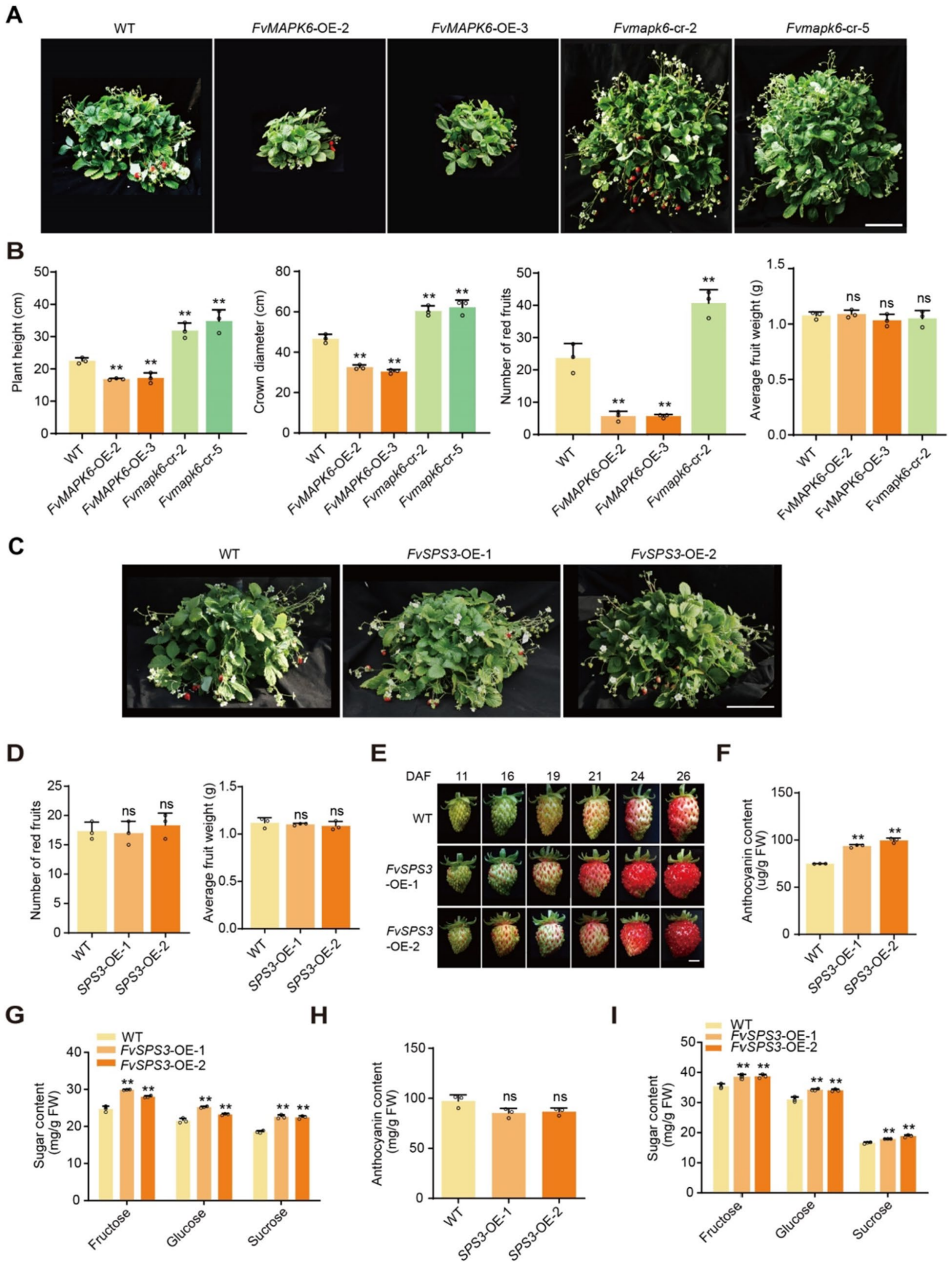
*FvMAPK6* regulates vegetative growth and yield in strawberry and *FvSPS3* regulates the anthocyanin and sugar content of strawberry fruits without impairing plant growth and yield. (A) Representative photographs of WT, *FvMAPK6*‐OE‐2, OE‐3 and *Fvmapk6‐*cr‐2, cr‐5 plants after 6 months of growth in the greenhouse (scale bar, 16 cm). (B) Plant height, crown diameter, and red fruit yield during the high production period in the indicated genotypes. (C) Representative photographs of WT, *FvSPS3‐*OE‐1 and OE‐2 plants after 6 months of growth in the greenhouse. Scale bar, 16 cm. (D) The yield of WT and *FvSPS3*‐OE, measured by the number and average fruit weight of red fruits, was evaluated. (E) Representative photographs of WT and *FvSPS3*‐OE‐1, OE‐2 fruits at different DAF. Scale bar, 0.7 cm. (F, G) Anthocyanin (F) and sugar contents (G) in WT and *FvSPS3*‐OE‐1, OE‐2 fruits at 26 DAF. (H, I) Anthocyanin (H) and sugar (I) contents in red fruits of WT and *FvSPS3*‐OE‐1, OE‐2; anthocyanin content was employed as a reference to evaluate the consistency of ripening degree between WT and *FvSPS3*‐OE‐1, OE‐2 fruits. Values are means ± SD. (*n* = 3 independent biological replicates 15 fruits per replicate in F–I; *n* = 3 plants in B, D). Significance was determined using Student's *t*‐test (two‐sided; *p < 0.05, **p < 0.01, ns, no significance).

While overexpressing *FvMAPK6* significantly enhanced sugar content in strawberry fruit, it resulted in undesirable plant dwarfing and reduced yield (Figure 1; Figure 6A,B). In contrast, *Fvmapk6‐cr* biallelic mutant plants exhibited enhanced growth and yield compared to WT plants (Figure 6A,B). These opposing phenotypes highlight the pleiotropic role of FvMAPK6 in mediating a trade‐off between growth/yield and fruit quality, indicating that direct modulation of *FvMAPK6* expression is unsuitable for breeding strategies focused exclusively on improving fruit quality.

To circumvent this trade‐off, we screened downstream components within the FvMAPK6 regulatory network that might possess more specialised functions in fruit ripening. Given that sucrose accumulation is a primary determinant of fruit sweetness across strawberry cultivars (Lee et al. 2018; Liu et al. 2020), we prioritised targets with direct or close functional associations with this process. An ideal candidate would be a direct target of the pathway with high expression in fruit and functionally dedicated to sucrose accumulation. Among the key enzymes involved, SPS catalyses a committed and unidirectional step in sucrose biosynthesis, unlike SUS and invertases (INV), which exhibit bidirectional roles in sucrose metabolism (Ren et al. 2023). Therefore, we focused on *FvSPS3*, the SPS family member regulated by the FvMAPK6‐FvMYB44 pathway (Figure 1; Figure S5) and exhibiting the highest fruit expression (Figure S10A,B). We hypothesised that *FvSPS3* could be a precise target for enhancing sugar content without the pleiotropic effects associated with manipulating the upstream kinase.

To test this, we generated *FvSPS3*‐overexpression (*FvSPS3*‐OE) lines. These lines exhibited normal plant morphology and fruiting characteristics (Figure S10C,D; Figure 6C–E), demonstrating that the overexpression of *FvSPS3* did not incur the growth penalties seen with *FvMAPK6* overexpression. Notably, anthocyanin and sugar accumulation were accelerated in *FvSPS3*‐OE fruits (Figure 6F,G). In commercial production, anthocyanin content is commonly utilised as a criterion for determining fruit ripeness at harvest. Therefore, we compared the sugar contents of WT and *FvSPS3*‐OE fruits with equivalent anthocyanin levels by adjusting the harvest time. Notably, at commercial maturity (defined by equivalent anthocyanin content), *FvSPS3*‐OE fruits accumulated significantly higher sugar levels than WT fruits (Figure 6H,I). This result indicates that *FvSPS3* not only accelerates ripening but also ultimately elevates the sugar content at the point of harvest. Collectively, these findings position *FvSPS3* as a highly promising and specific target for the genetic improvement of strawberry fruit quality.

## 3 Discussion

The MAPK kinase family plays crucial roles in signal transduction in both animals and plants (Zhang and Zhang 2022). Although the functions of MAPKs in horticultural crops have been explored (Yang et al. 2021; Mao et al. 2022; Xing et al. 2023), their specific roles in regulating sugar accumulation remain unclear in both horticultural and other plant species. Here, we provide the first genetic evidence that FvMAPK6 directly regulates the accumulation of endogenous sucrose, glucose and fructose in fruits (Figure 1). Notably, we also found that the overexpression of *FvMAPK6* improves fruit quality, including both sugar and anthocyanin accumulation, but concurrently leads to a significant yield penalty, primarily due to reduced plant size and fewer fruiting branches (Figure 6A,B). This trade‐off between quality and yield presents a key challenge in crop improvement and exhibits species‐specificity. For instance, while MAPK6 activation enhances grain size and weight in rice (Xu et al. 2018; Bai et al. 2024), thereby increasing yield, our results demonstrate that FvMAPK6 negatively regulates yield in strawberry. This functional divergence highlights the species‐specificity of MAPK6‐mediated regulation. The substantial yield increase observed in *fvmapk6‐cr* biallelic mutants, despite delayed ripening, highlights the potential of modulating this pathway to enhance strawberry productivity (Figures 1 and 6B). These findings suggest that MAPK6 may serve as a conserved regulator of yield‐related traits, yet its mechanistic actions and phenotypic outcomes are species‐specific.

To decouple high‐quality traits from the yield penalty, we targeted downstream components of the FvMAPK6 pathway. By overexpressing *FvSPS3*, we successfully developed a new strawberry germplasm with enhanced fruit quality without compromising yield. The significance of *FvSPS3* is further supported by its prior identification as a candidate gene for a sugar‐related marker through *cis*‐eQTL analysis (Fan et al. 2024; Prohaska et al. 2024), aligning with our functional findings (Figure 6D–I). Thus, our study not only establishes FvMAPK6 as a key regulator in a sucrose‐signalling network but also provides a viable strategy to overcome the yield‐quality trade‐off. Notably, applying base editor‐mediated genome editing to octoploid cultivated strawberry cultivars for targeted modification of the *FvSPS3* gene holds great promise: this approach could generate early‐maturing, high‐sugar strawberry cultivars that maintain yield, thereby offering significant potential for industrial application in strawberry production (Xing et al. 2020). Additionally, further systematic analysis of the FvMAPK6‐mediated regulatory mechanism will facilitate the development of more precise breeding strategies aimed at synergistically improving both yield and quality in strawberry.

**FIGURE 7:**
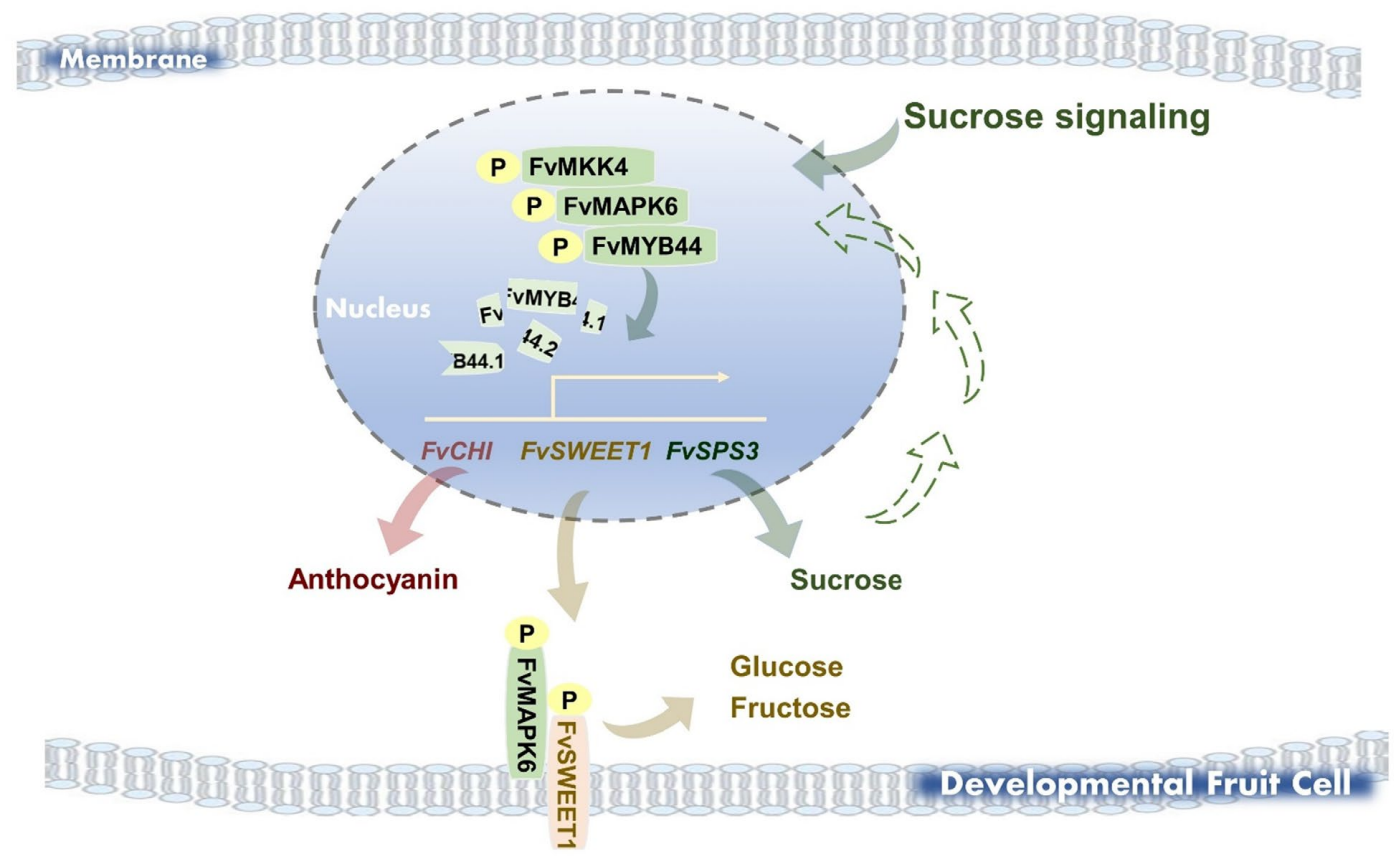
Model of how sucrose‐activated FvMAPK6 coordinates sugar and anthocyanin accumulation in strawberry fruit. During strawberry fruit development, FvMAPK6 exhibits a high level of phosphorylation, which is further enhanced by sucrose treatment. Specifically, sucrose‐activated FvMAPK6 forms a phosphorylation cascade with FvMKK4 that directly phosphorylates the transcriptional repressors FvMYB44.1 and FvMYB44.2. This phosphorylation attenuates their repressive function by promoting protein degradation and reducing transcriptional activity, thereby derepressing sugar accumulation and anthocyanin biosynthesis. Additionally, FvMAPK6 increases the protein abundance and phosphorylation‐mediated transport activity of the hexose transporter FvSWEET1, which further enhances sugar accumulation. In summary, FvMAPK6 acts as a sucrose‐responsive kinase that coordinates sugar and anthocyanin accumulation via a dual mechanism: (1) a transcriptional pathway mediated by direct phosphorylation of FvMYB44.1/44.2, and (2) a post‐translational pathway mediated by FvSWEET1 phosphorylation. Solid arrows represent regulatory relationships established in this study; dashed arrows indicate putative mechanisms based on current and prior evidence. The potential mechanism by which FvMAPK6‐generated endogenous sucrose functions as a signalling molecule to directly amplify downstream biological events remains to be elucidated in future studies.

Building on previous work that identified FvMYB44.2 as a repressor of sucrose accumulation (Wei et al. 2018), our study identifies FvMAPK6 as its upstream kinase (Figure 2). Moreover, we demonstrate that FvMYB44.1 also contributes to the coordinated regulation of sugar and anthocyanin (Figure S5), implying co‐regulation of sucrose accumulation with the FvMYB44 family. Although MYB44 proteins regulate carbohydrate accumulation in diverse plants (Li et al. 2021; Gao, Yang, et al. 2023; Wang et al. 2024; Liu et al. 2025), their upstream regulators have remained elusive. Our study bridges this gap by demonstrating that the FvMAPKK4–FvMAPK6 cascade—previously not associated with sugar metabolism—directly phosphorylates FvMYB44.1/44.2, thereby reducing their stability and transcriptional activity (Figures 3 and 7). This phosphorylation attenuates the repression by FvMYB44s, thus modulating downstream genes involved in anthocyanin biosynthesis, sugar metabolism and transport, including *FvSPS3* and *FvSWEET1*(Figure 3E,F). This mechanism provides a foundational framework for understanding MYB44‐mediated carbohydrate regulation across species. The functional conservation of MYB44 is highlighted by recent studies in wheat and rice, where it regulates grain starch content, and its knockout increases starch accumulation and grain weight (Liu et al. 2025). These collective findings position MYB44 as a promising target for enhancing carbohydrate‐related traits in a broad range of crops.

Fruit ripening is not solely governed by sucrose signalling but is also influenced by ABA, auxin, and environmental factors such as light and temperature (Durán‐Soria et al. 2020). Notably, FvMAPK6 also significantly affected the phosphorylation of crucial components involved in these signalling pathways, such as ASR, PIN1 and PHOTOTROPINs (PHOTs) (Figure S6). Furthermore, the phosphorylation of FvKIN10 was also significantly regulated by FvMAPK6 (Figure S6). KIN10 (SnRK1α) serves as a central regulator in plant responses to sugar deprivation triggered by environmental stresses. It modulates both key enzymes involved in sugar metabolism and bZIP family transcription factors through phosphorylation, thereby mediating carbon reallocation under adverse conditions (Li and Sheen 2016). In *Arabidopsis*, ABA activates SnRK1α, leading to the phosphorylation of RAPTOR and subsequent inhibition of TOR activity during the drought response (Han et al. 2023). In addition, light signals repress the activity of SnRK1α in regulating circadian rhythms in *Arabidopsis* (Han et al. 2023). Both light and ABA serve as regulatory signals that facilitate strawberry fruit ripening and quality formation (Kadomura‐Ishikawa et al. 2015). Notably, the transient overexpression of *FvSnRK1α* in strawberry fruits accelerates fruit ripening (Luo et al. 2020). These results collectively suggest that FvMAPK6 may serve as a central phosphorylation hub that facilitates multiple signal transduction pathways and crosstalk during strawberry fruit ripening and quality regulation.

SWEET proteins are key sugar transporters in plants, with different members exhibiting distinct substrate specificities (Chen et al. 2010). In *Arabidopsis*, for example, SnRK2 kinases regulate the oligomerisation and transport activity of the sucrose transporters AtSWEET11 and AtSWEET12 through phosphorylation (Chen et al. 2022). Here, we report a distinct regulatory mechanism, demonstrating that FvMAPK6 enhances hexose transport activity of FvSWEET1 through phosphorylation of 223S in its C‐terminal region (Figure S7). Consistent with its regulatory role, fruit‐specific overexpression of *FvSWEET1* significantly increases sugar content (Figure 5F,G). The identification of MAPK‐ and SnRK2‐mediated phosphorylation pathways targeting different SWEET members raises the intriguing possibility that these kinase families represent parallel, specialised regulatory modules for fine‐tuning sugar allocation in plants. Notably, the regulation of FvSWEET1 by FvMAPK6 is amplified by sucrose signalling (Figure 5D,G). This indicates that sugar molecules, even those not transported by a specific transporter, can modulate its activity through signalling cascades, highlighting an intricate interplay between sugar signalling and transport. Our findings not only expand the mechanistic understanding of SWEET protein regulation but also demonstrate a direct link between a sucrose‐activated MAPK cascade and hexose transport (Figure 7). While this study focuses on the role of FvSWEET1 in fruit sugar accumulation, a comprehensive understanding of its physiological function will require future investigation into its contribution to long‐distance source‐to‐sink transport.

Beyond the detailed mechanisms involving FvMYB44s and FvSWEET1, our phosphoproteomic analysis of FvMAPK6 identified additional components with reported roles in sugar biosynthesis, metabolism and signalling, supporting its role as a central phosphorylation hub (Figure S6). Interestingly, the phosphorylation levels of homologues of the tomato ‘sugar brakes’ SlCDPK26 and SlCDPK27 were also significantly modulated by FvMAPK6 (Zhang et al. 2024, Figure S6A). Given that sucrose is a key quality determinant in strawberry fruits but not in tomato, which has extremely low sucrose content, the potential conservation of a FvMAPK6‐mediated sugar accumulation pathway across both climacteric and non‐climacteric fruits is particularly intriguing (Lee et al. 2018; Liu et al. 2020; Zhang et al. 2024). This suggests that modulating MAPK6 activity to enhance sugar content in tomato and other fruit crops is a promising research direction. Previous studies also have demonstrated that MAPK6 plays a regulatory role in ethylene biosynthesis and signalling transduction (Liu and Zhang 2004; Zhou et al. 2022). Ethylene serves as the central regulator of ripening in climacteric fruits such as tomato, which exhibit a respiratory burst during maturation. In contrast, ripening in non‐climacteric fruits like strawberry is primarily governed by abscisic acid (ABA) (Li et al. 2022). Therefore, a comparative analysis of how MAPK6 modulates ripening in climacteric versus non‐climacteric species is highly warranted. Moreover, the identification of key signalling components like FvSnRK1α as phosphorylation targets in our dataset further suggests the central role of FvMAPK6 in sugar signalling networks and warrants future investigation (Figure S6B).

## 4 Experimental Procedures

### 4.1 Plant Materials and Growth Conditions

The plant materials used in this study were octoploid strawberry (*Fragaria × ananassa* Duch. cv. Benihoppe), diploid strawberry ( *Fragaria vesca* cv. Ruegen) and *Nicotiana benthamiana*. Strawberry plants were cultivated under controlled greenhouse conditions or in a growth chamber at 25°C, with a relative humidity of 40%–60%, under a 12 h light/12 h dark photoperiod, and an irradiance level of 200–300 μmol m^−2^ s^−1^ using white fluorescent T5 tubes (14 W). *N. benthamiana* plants were grown exclusively in the growth chamber.

### 4.2 Stable Transformation of Diploid Strawberry

The full‐length coding sequence of *FvMAPK6* without the termination codon, which includes a sequence encoding an HA‐tag at the C terminus, was cloned into the Gateway donor vector pDONR221 using Gateway BP Clonase (Invitrogen, 11 789 020) and confirmed by sequencing. It was then transferred into the destination vector pH7WG2D using Gateway LR Clonase (Invitrogen, 11791020) to generate the *FvMAPK6*‐OE vector. Genome editing was performed using the CRISPR/Cas9 vector pYLCRISPR/Cas9, as previously described (Zeng et al. 2018). To generate knockouts of *FvMAPK6*, specific regions within the coding sequence at the positions 25–44 bp, 97–116 bp and 366–384 bp were selected as target sites and their corresponding single guide RNAs (sgRNAs) were cloned into expression cassettes driven by the *AtU6‐29*, *AtU3d* and *AtU3b* promoters respectively. The resulting expression cassettes were incorporated into the pYLCRISPR/Cas9Pubi‐H binary plasmid using Golden Gate ligation. The final vectors were transformed into Agrobacterium ( *Agrobacterium tumefaciens* ) strain GV3101 for the stable transformation of diploid strawberry cv. ‘Ruegen’ plants as previously described (Mao et al. 2022). The overexpressing plants were detected by RT‐qPCR, and immunoblotting with anti‐Phospho‐p44/42 MAPK (Erk1/2) (Thr202/Tyr204) (Cell Signalling Technology, 4370S, 1:1000 dilution) and anti‐HA antibody (ABclonal, AE008, 1:1000 dilution). The gene‐edited strawberry plants were identified by DNA sequencing and a kinase activity assay with anti‐phospho‐p44/42 antibody. The primers used are listed in Table S4.

### 4.3 Transient Expression Assays in Strawberry Fruits

To examine the function of *FvMYB44.1*, *FvSWEET1* and *FvSWEET1* ^*D*^, the full‐length coding sequence of *FvMYB44.1, FvSWEET1* and *FvSWEET1* ^*D*^ was cloned into the pH7WG2D vector by Gateway cloning to obtain the overexpression vector (OE), while two specific fragments of the *FvMYB44.1* coding sequence were inserted into pFGC5941 to obtain the *FvMYB44.1*‐RNAi vector. Agrobacterium‐mediated transient infiltration of strawberry fruits was performed as previously described (Wei et al. 2018). To identify the regulatory signalling pathways in strawberry fruits, a GUS activity assay was performed based on transient expression. The promoters of *FvCHS*, *FvCHI*, *FvSPS3*, *FvSWEET1* and *FvSS2* were cloned in place of the cauliflower mosaic virus (CaMV) 35S promoter in the pCAMBIA1301 vector to obtain different reporter constructs. The full‐length coding sequences of *FvMAPK6* and *FvMYB44.1*/*44.1* ^*A/D*^/*44.2/44.2* ^*A/D*^ were cloned into the pBI121 vector and placed under the control of the 35S promoter to generate effectors constructs. The effects of different effectors on GUS activity were evaluated via Agrobacterium‐mediated infiltration of strawberry fruits. At 24 h post‐infiltration, the fruits were transferred to 50 mM sucrose or water only, incubated for 24 h, and frozen in liquid nitrogen for a GUS activity assay as previously described (Wei et al. 2018). The primers used are listed in Table S4.

### 4.4 Measuring Anthocyanin and Sugar Contents

Sugar content in strawberry fruits was measured using HPLC as previously described (Kafkas et al. 2007). Anthocyanins in strawberry fruits were extracted and quantified using a Plant Anthocyanin Content kit (COMIN, HSG‐1‐Y), as previously described (Chen et al. 2023).

### 4.5 RNA Isolation and Reverse‐Transcription Quantitative PCR (RT‐qPCR)

Total RNA was extracted from strawberry tissues using an E.Z.N.A. Total RNA kit (Omega) according to the manufacturer's instructions. RT‐qPCR was performed as previously described (Mao et al. 2022). *FvACTIN* (FvH4_6g22300) served as the internal reference gene. Three biological replicates were analysed, and relative expression levels were determined using the delta–delta cycle threshold method (2^–ΔΔCT^). The primer sequences used for RT‐qPCR are provided in Table S4.

### 4.6 Transcriptome Assay

Total RNA was extracted from WT, *FvMAPK6*‐OE and *Fvmapk6*‐cr red fruits using an E.Z.N.A. Total RNA kit (Omega, Bienne, Switzerland). Sequencing was performed on an Illumina NovaSeq instrument and aligned to the *F. vesca* spp. *vesca* reference genome (https://www.rosaceae.org/rosaceae_downloads/Fragaria_vesca/Fvesca‐genome.v4.0.a1/assembly/Fragaria_vesca_v4.0.a1.fasta.gz) by novogene. Genes showing significant differential expression were identified based on an adjusted *p*‐value < 0.05 and |log_2_(fold‐change) | > 1 using the DESeq2. This experiment included three independent biological replicates.

### 4.7 Recombinant Protein Production and Purification

The full‐length coding sequences of *FvMAPK6*, *FvMYB44.1* and *FvMYB44.2* were cloned into the pET30a vector, while the full‐length coding sequences of *FvMAPKK4* and *FvMAPK6* were cloned into the pGEX6P‐1 vectors. Point mutations T227D/S233D (DD) were introduced into FvMKK4 (FvMKK4^DD^), mutations 62A were introduced into FvMYB44.1 (FvMYB44.1^A^) and mutations 226A were introduced into FvMYB44.2 (FvMYB44.2^A^) using PCR amplification techniques. *Escherichia coli* BL21 (DE3; Transgene) was used for plasmid transformation to express recombinant proteins, which were subsequently purified using Glutathione Sepharose beads (Cytiva, 17–0756‐01) and Ni Sepharose 6 Fast Flow Resin (Cytiva, 17–5318‐01). The primers used are listed in Table S4.

### 4.8 Electrophoretic Mobility Shift Assay (EMSA)

The cis‐acting elements in the promoter regions of *FvSPS3*, *FvSS2*, *FvSWEE1*, *FvCHS1* and *FvCHI* were analysed using Plant CARE (http://bioinformatics.psb.ugent.be/webtools/plantcare/html/). Oligonucleotide probes were synthesised and labelled with biotin at the 5′ end by DIA‐UP BIOTECH. The recombinant FvMYB44.1‐His proteins were produced in *E. coli* BL21 cells. EMSA was performed using a Chemiluminescent EMSA kit (Beyotime, GS009) according to the manufacturer's instructions. The EMSA probe sequences can be found in Table S4.

### 4.9 Protein Extraction and Immunoblotting

Protein extraction and immunoblotting were performed as previously described (Mao et al. 2022). Briefly, strawberry fruits or leaves were ground into a powder in liquid nitrogen. Protein extraction was carried out using extraction buffer consisting of 100 mM HEPES buffer (pH 7.2–7.4), 1 mM EDTA, 10% (v/v) glycerol, 1% (v/v) Triton X‐100, 1 mM DTT, and protease/phosphatase inhibitor cocktails (CWBIO, CW2200/CW2383) (both at a final concentration of 1×). The extracted proteins were used to measure the abundance or phosphorylation levels of specific proteins. Commercially available antibodies were used for immunoblotting, including anti‐His (CWBIO, CW0286, 1:1000 dilution), anti‐GST (CWBIO, CW0084, 1:1000 dilution), anti‐HA (ABclonal, AE008, 1:1000 dilution), anti‐AtMAPK6 (SIGMA, A7104, 1:1000 dilution), anti‐Phospho‐p44/42 MAPK (Erk1/2) (Thr202/Tyr204) (Cell Signalling Technology, 4370S, 1:1000 dilution) and anti‐β‐actin (ABclonal, AC009, 1:1000 dilution). The anti‐FvMYB44.1 antibody was generated by ABclonal, while the anti‐FvSWEET1 antibody was produced by Abmart. Specific polyclonal antibodies were raised in rabbits using the peptides C‐GNGVGYDGNFHEQP and C‐YRDIKKQQPSAEDSVELGLEKPHQSKQANGVNGV, which were selected based on the C terminus of FvMYB44.1 and FvSWEET1, respectively. Recombinant FvMYB44.1‐His and FvSWEET‐C‐GST proteins produced in *E. coli* BL21(DE3) cells were employed to assess antibody specificity.

### 4.10 Protein Interaction Assays

#### 4.10.1 Yeast Two‐Hybrid (Y2H) Assay

The full‐length coding sequence of *FvMAPK6* was cloned into the pGADT7 vector, and the full‐length coding sequences of *FvMAPKK*s were individually cloned into the pGBKT7 vector. The resulting constructs were introduced into yeast strain AH109 according to the manufacturer's instructions (WEIDI, CAT#: YC1010). Yeast colonies were selected on synthetic defined (SD) medium lacking Leu and Trp (−L − W), transferred to SD medium lacking Leu, Trp, His, and Ade (‐Leu‐Trp‐His‐Ade), and incubated at 28°C for 3 days before assessing colony growth.

#### 4.10.2 Bimolecular Fluorescence Complementation (BiFC) Assay

The full‐length coding sequence of *FvMAPK6* was cloned into the pSPYNE vector, while the full‐length coding sequences of *FvMYB44.1/44.2* and *FvSWEET1* were individually cloned into the pSPYCE vector. The vectors were individually transformed into Agrobacterium strain GV3101; the appropriate pairs of Agrobacterium cultures were resuspended in infiltration buffer (10 mM MES pH 5.6, 10 mM MgCl_2_ and 200 μM acetosyringone), mixed in a 1:1 (v/v) ratio, and co‐infiltrated into the leaves of 4–6‐week‐old *N. benthamiana* plants. Following 48–72 h of incubation in the dark, YFP fluorescent signals were visualised under a confocal laser‐scanning microscope (Leica SP8).

#### 4.10.3 Luciferase Complementation Imaging (LCI) Assay

The full‐length coding sequence of *FvMAPK6* was cloned into the pCAMBIA1300‐nLUC vector, while the full‐length coding sequences of *FvMYB44.1/44.2* and *FvSWEET1* were cloned into the pCAMBIA1300‐cLUC vector. The resulting constructs were individually transformed into Agrobacterium strain GV3101. Positive agrobacterium strains containing the appropriate nLUC or cLUC vector were resuspended in infiltration buffer and mixed in a 1:1 (v/v) ratio before the leaves or fruits were infiltrated with cell suspension. After 48–72 h of culture in the dark, luciferase activity was assessed using Lumazone and WinView software.

#### 4.10.4 Pull‐Down Assay

The recombinant plasmids, pET30a‐*FvMYB44.1/MYB44.2* and pGEX‐6P‐1*‐FvMAPK6*, were transformed into *E. coli* BL21 (DE3) cells. The supernatant containing FvMYB44.1/44.2‐His protein was mixed separately with the supernatant containing FvMAPK6‐GST or GST and incubated with Ni Sepharose 6 Fast Flow Resin (Cytiva, 17–5318‐01) at 4°C for 2 h. The FvMYB44.1/44.2‐His and GST or FvMYB44.1/44.2‐His and FvMAPK6‐GST complexes were eluted from the Ni‐NTA agarose beads and analysed by immunoblotting.

#### 4.10.5 Co‐Immunoprecipitation (Co‐IP) Mass Spectrometry (MS) Assay

Total proteins were extracted from WT and *FvMAPK6*‐OE red fruits as previously described and incubated overnight with anti‐FvMAPK6 antibody (SIGMA, A7104) at 4°C. After adding 40 μL of protein A + G agarose (Beyotime, P2012), the samples were incubated at 4°C for 2 h. The agarose beads were washed three to five times with extraction buffer. After adding 40 μL of SDS loading buffer, the samples were vortexed and incubated in a boiling water bath for 5 min. The samples were subjected to SDS‐PAGE and analysed by LC–MS/MS.

#### 4.10.6 Pull‐Down MS Assay

Total proteins were extracted from WT fruits at the turning fruit stage as described above. The protein extracts were incubated with recombinant purified His‐FvMYB44.2 in the presence of Ni Sepharose 6 Fast Flow (Cytiva, 17‐5318‐01) at 4°C for 10 h. The sample was washed three to four times with protein extraction buffer, mixed with SDS loading buffer, and boiled. The samples were subjected to SDS‐PAGE and analysed by LC–MS/MS.

### 4.11 Measuring Protein Phosphorylation

#### 4.11.1 In Vitro Kinase Assay

Recombinant FvMKK4^DD^‐GST and FvMAPK6‐His proteins were incubated in reaction buffer (20 mM Tris–HCl, pH 7.5, 10 mM MgCl_2_, 1 mM DTT and 50 mM ATP) at 30°C for 30 min to activate FvMAPK6‐His. The activated FvMAPK6‐His was incubated with FvMYB44.1 or MYB44.1^A^ or MYB44.2 or MYB44.2^A^‐His in kinase reaction buffer (20 mM Tris–HCl, pH 7.5, 10 mM MgCl_2_, 1 mM DTT, 25 mM ATP and [γ32p] ATP at a concentration of 1 μCi) at 30°C for 30 min. The reaction was terminated by adding SDS loading buffer, and the samples were subjected to SDS‐PAGE. The gel was exposed to a phosphor imager screen for approximately 24–48 h and visualised using a Typhoon9410 imager.

#### 4.11.2 Phos‐Tag Assay

The phosphorylation of FvSWEET1 in WT, *FvMAPK6*‐OE and *Fvmapk6*‐cr red fruits was assessed using a 10% (w/v) SDS‐PAGE gel containing 50 μM phos‐tag reagent (Wako‐chem, AAL‐107) and 100 μM MnCl_2_. After sample separation, the gel was incubated at 30°C for 20 min with or without Lambda Protein Phosphatase (Beyotime, P2316S) to detect phosphorylation modification. Finally, FvSWEET1 and its phosphorylated form were detected by probing with anti‐FvSWEET1 antibody and analysis of molecular weight.

#### 4.11.3 4D‐FastDIA Quantitative Phosphoproteomics

Red fruits were collected from WT, *FvMAPK6*‐OE and *Fvmapk6*‐cr plants in two biological replicates, with each replicate consisting of 15 fruits. A phosphoproteomics assay was conducted by PTM‐BIO Company. The protein samples were enzymatically hydrolysed in equal quantities. Trypsin was added at a ratio of 1:50 (protease: protein, w/w), and the sample was digested overnight. The peptides were dissolved in enrichment buffer (50% [v/v] acetonitrile, 0.5% [v/v] acetic acid), and the supernatant was transferred to IMAC material and then incubated using a rotary shaker gently. After that, the sample was washed three times in buffer (50% [v/v] acetonitrile, 0.5% [v/v] acetic acid and 30% [v/v] acetonitrile, 0.1% [v/v] trifluoroacetic acid). The phosphopeptides were eluted with 10% (w/v) ammonia, and the eluent was collected and freeze‐dried. The salt was removed from the dried sample using C18 Zip Tips, and the sample was freeze‐dried for LC–MS. The data used in this experiment were retrieved from the Fragaria_vesca_57918_Rosaceae_GDR_ 20 200 619.fasta (64 597 sequences) file using the Spectronaut (v18) search engine with default parameters.

### 4.12 Cell‐Free Degradation Assays

The recombinant protein His‐FvMYB44.1/MYB44.2 was added to total proteins extracted from WT, *FvMAPK6*‐OE, and *Fvmapk6*‐cr fruits and were incubated in buffer containing 50 mM Tris‐MES pH 8.0, 500 mM sucrose, 1 mM MgCl_2_, 10 mM EDTA and 5 mM DTT. The reaction mixture was incubated at 37°C and sampled periodically for immunoblot analysis using an anti‐His antibody. To investigate the effect of ubiquitination on degradation, the proteasome inhibitor MG132 (50 μM) or DMSO was added to the protein mixture.

### 4.13 Assay of the Transport Characteristics of FvSWEET1s in Yeast

The full‐length coding sequences of *FvSWEET1* and its Ser 223 mutants *FvSWEET1* ^*D*^ (phosphomimetic mutant) and *FvSWEET1* ^*A*^ (phosphorylation‐deficient mutant) were individually cloned into the yeast expression vector pDR196; the resulting constructs were transformed into the hexose uptake mutant yeast ( *Saccharomyces cerevisiae* ) strain EBY.VW4000 or the hexose and sucrose uptake mutant yeast ( *Saccharomyces cerevisiae* ) strain CSY4000. The transformed cells were cultivated on yeast medium (Cat. PM2273‐5 L; Coolaber, Beijing, China) at 30°C for 2–3 days, and yeast growth was assessed as previously described (Cheng et al. 2015). The growth status was used to determine the transport substrate specificity and activity of FvSWEET1. The primers used are listed in Table S4.

### 4.14 Sugar Treatment of Strawberry Fruits

To evaluate the effect of sucrose on fruit ripening, white‐stage fruits were sprayed with a nutrient solution. The treatments involved supplementing the buffer with water, mannitol or sucrose. Phenotypic images were captured at various time points during the treatment. For molecular analyses, fruits treated for 2 h were used for immunoblotting, while those treated for 2 days were used for RT‐qPCR and determination of anthocyanin and sugar content. All samples were flash‐frozen in liquid nitrogen, and proteins were extracted as described above.

### 4.15 Statistical Analysis

Statistical analysis was performed by two‐tailed Student's *t*‐test using GraphPad Prism version 11.0 and one‐way analysis of variance (ANOVA) with Tukey's test using SPSS version 26.0. All graphs were generated using GraphPad Prism version 10.0.

## Author Contributions

B.L. and Q.F. designed the experiments, while Q.F. performed most of them. L.W., T.L. and K.W. assisted in generating the transgenic materials. X.L., C.L., R.S., X.L., Z.Y., Y.W., H.Y. and Q.L. aided in data analysis. B.L. and Q.F. wrote the article.

## Funding

This work was supported by the National Key Research and Development Program (2023YFF1001700), the National Natural Science Foundation of China (32222074, 32572990), the National Key Research and Development Program (2022YFD2100102‐3), Beijing Rural Revitalization Agricultural Technology Project (NY2401150024), the 2115 Talent Development Program of China Agricultural University and 111 Project (B17043). The yeast mutant strains EBY.VW4000 and CSY4000 were kindly provided by Prof. Dong Meng from Beijing Forestry University. We thank Prof. Zhen Fan for his assistance in analyzing the sugar‐related markers.

## Conflicts of Interest

The authors declare no conflicts of interest.

## Supporting information


**Figure S1:** Chromatogram revealing the binding peptide of FvMAPK6, identified via pull‐down MS using recombinant FvMYB44.2‐GST.
**Figure S2:** Generation of *FvMAPK6* overexpression and gene‐edited strawberries. (A) *FvMAPK6* expression levels in *FvMAPK6*‐OE lines (*FvMAPK6*‐OE‐2 and *FvMAPK6*‐OE‐3) using RT‐qPCR. (B) Kinase activity of FvMAPK6 in *FvMAPK6*‐OE red fruits, as detected using anti‐p44/42 antibody. (C) Diagram of the *FvMAPK6* locus and target sites of the sgRNAs. The target sequences located at positions 25–44 bp, 97–116 bp and 366–384 bp within the coding sequence of *FvMAPK6* were selected for CRISPR/Cas9‐mediated gene editing. Green boxes, exons; lines, introns. Two plants, biallelic *Fvmapk6*‐cr‐2 (with a 3‐bp deletion and a 5‐bp deletion for each allele) and *Fvmapk6*‐cr‐5 (homozygous for a 5‐bp deletion). In the T2 generation, a new biallelic plant (*Fvmapk6*‐cr‐2‐1) was identified with different edits compared to *Fvmapk6*‐cr‐2: one allele had a 5‐bp deletion while the other allele had a substitution of 1 bp along with a three‐bp deletion. (D) Kinase activity is decreased in *Fvmapk6*‐cr‐2 red fruits and completely abolished in *Fvmapk6*‐cr‐5 leaves. (E) *Fvmapk6*‐cr‐5 exhibits abnormal fertility and no fruit production. Scale bar, 6 cm. (F‐I) Validation of the biological function of FvMAPK6 in *Fvmapk6*‐cr‐2‐1 fruits by assessing fruit ripening (F), anthocyanin content (G), sugar content (H) and gene expression level (I) at 26 DAF. Scale bar, 0.6 cm. Values are means ± SD. in A (*n* = 3 biological replicates; each replicate contained ten leaves) and G, H, I (*n* = 3 biological replicates; each replicate contained 15 fruits). Statistical significance was determined by Student's *t*‐test (two‐sided, *p < 0.05, **p < 0.01, ns, no significance).
**Figure S3:** Identification of the FvMAPKK4–MAPK6 phosphorylation cascade. (A) Yeast two‐hybrid (Y2H) assay to detect interactions between FvMAPK6 and FvMAPKKKs. The fusion vectors pGBKT7(BD)‐FvMAPKK1‐9 and pGADT7(AD) or pGADT7(AD)‐FvMAPK6 were co‐transformed into yeast cells, followed by growth on selection medium. (B) Mass spectra of FvMAPKK4 peptides in *FvMAPK6*‐OE fruits obtained by immunoprecipitation followed by mass spectrometry. (C) Expression pattern of fruit‐expressed *FvMAPKK*s in ‘Ruegen’ fruits at different developmental stages, as revealed by RNA‐seq data. BG, big green fruit; W, white fruit; T, turning fruit; R, red fruit.
**Figure S4:** Identification of the phosphorylated amino acids in FvMYB44.1 and FvMYB44.2 using LC–MS/MS.
**Figure S5:** FvMYB44.1 directly regulates the expression of *FvCHS1*, *FvCHI*, *FvSWEET1*, *FvSPS3* and *FvSS2*. (A) Representative photographs of transiently overexpressing (*FvMYB44.1*‐OE) and *FvMYB44.1*‐silenced (*FvMYB44.1*‐RNAi) ‘Benihoppe’ strawberry fruits. DAI, days after infiltration. Scale bar, 1 cm. (B, C) RT‐qPCR (B) and Western blot (C) analyses of FvMYB44.1 expression in *FvMYB44.1*‐OE and *FvMYB44.1*‐RNAi fruits. (D, E) Anthocyanin (D) and sugar (E) contents in *FvMYB44.1*‐OE and *FvMYB44.1*‐RNAi fruits at 7 DAI. (F) Relative expression levels of genes involved in anthocyanin biosynthesis and sugar metabolism in *FvMYB44.1*‐OE and *FvMYB44.1*‐RNAi fruits, are quantified by RT‐qPCR; *FvACTIN* served as the internal reference gene. (G) In vitro electrophoretic mobility shift assay (EMSA) demonstrating the binding affinity of recombinant FvMYB44.1‐His to the promoters of *FvCHS1*, *FvCHI*, *FvSWEET1*, *FvSPS3* and *FvSS2* following incubation of biotin‐labelled probes and different concentrations of unlabeled probes followed by PAGE. (H) GUS activity assay revealing that overexpressing *FvMYB44.1* suppresses of the expression of *FvCHS1, FvCHI*, *FvSWEET1*, *FvSPS3* and *FvSUS2*. The *GUS* reporter gene was driven by each indicated promoter (*FvCHS1*, *FvCHI*, *FvSWEET1*, *FvSPS3*, *FvSUS2*). Transient co‐infiltration experiments were conducted using ‘Benihoppe’ fruits with the *35S:FvMYB44.1* construct and the indicated *GUS* construct. Values are means ± SD. (*n* = 3 independent biological replicates; each replicate contained 15 fruits). Significance was determined using Student's *t*‐test (two‐sided, *p < 0.05, **p < 0.01, ns, no significance).
**Figure S6:** FvMAPK6 modulates the phosphorylation status of proteins involved in the SnRK1‐TOR network as well as sugar transport, metabolism and signal transduction, as revealed by FvMAPK6‐related phosphoproteomics. (A, B) Heatmap representation of changes in phosphorylation of proteins associated with sugar transport, metabolism (A) and SnRK1‐TOR network (B). The identification of related proteins in strawberries as presented in B, was based on the research progress made in other plant species. Specific members were determined through homology analysis (Huang et al. 2014; Cho et al. 2016; Shukla et al. 2018; Jamsheer et al. 2021; Van Leene et al. 2022; Gao, Zhang, et al. 2023; Liao et al. 2023).
**Figure S7:** Identification of FvSWEET1 phosphorylation sites mediated by FvMAPK6 via phosphoproteomics.
**Figure S8:** The expression pattern of *SWEET* gene in strawberry and the phylogenetic tree analysis of SWEET in strawberry, *Arabidopsis thaliana* and rice. (A, B) Heatmap representation of the expression levels for *FvSWEET*s based on RNA‐seq data obtained from the fruits at different stages of development in ‘Ruegen’ strawberry (A) and the red fruits of WT, *FvMAPK6*‐OE and *Fvmapk6*‐cr (B). G, green fruit; W, white fruit; T, turning fruit; R, red fruit; each number indicates a different replicate. The colour scale represents the log_2_ FPKM values. (C) Phylogenetic tree of FvSWEETs with Arabidopsis and rice orthologs reconstructed using the neighbour‐joining method with MEGA version 11.0. FvSWEET1 is indicated by an asterisk (*).
**Figure S9:** Expression levels of *FvCHI*, *FvSPS3* and *FvSWEET1* in sucrose‐treated fruits of WT and *FvMAPK6* transgenic lines determined by RT‐qPCR. WT and *FvMAPK6*‐OE fruits (A) or WT and *Fvmapk6*‐cr fruits (B) were treated at the white fruit stage, with samples collected 2 days post‐treatment. CK1, white fruit treated with water; CK2, white fruit treated with 50 mM mannitol; Suc, white fruit treated with 50 mM sucrose. Values are means ± SD. (*n* = 3 independent biological replicates); each replicate contained 10 fruits. Different lowercase letters indicate significant differences, as assessed by one‐way ANOVA (with Tukey's test) (*p* < 0.05).
**Figure S10:** Generation and phenotypic analysis of *FvSPS3*‐OE plants. (A, B) *FvSPS* expression levels in ‘Ruegen’ leaves (A) and fruits (B), as determined by RNA‐seq data. BG, big green fruit; W, white fruit; T, turning fruit; R, red fruit. (C) *FvSPS3*‐OE transgenic plants were identified by RT‐qPCR. (D) Representative photographs of WT and *FvSPS3*‐OE plants. Scale bar, 8 cm. Values are means ± SD. (*n* = 3 independent biological replicates; each replicate contained 10 leaves) in c. Significance was determined using Student's *t*‐test (two‐sided, *p < 0.05, **p < 0.01).
**Table S1:** Identification of specific FvMYB44.2‐binding proteins by Pull down‐MS (Partial Data).
**Table S2:** Selected differentially expressed genes (DEGs) in *Fvmapk6*‐cr fruits compared to WT fruits.
**Table S3:** Identifying proteins involved in the accumulation of anthocyanin and sugar using IP‐MS with anti‐MAPK6 (Partial Data).
**Table S4:** Primers used in this study.

## Data Availability

The data that support the findings of this study are available on request from the corresponding author.
