# Supplementary material for: FvMAPK6‐Mediated FvMYB44s/FvSWEET1 Dual‐Layer Regulation Modulates Sugar Accumulation in Strawberry Fruit, With FvSPS3 Enabling Quality–Yield Balance
Source: Plant Biotechnol J. 2026 Mar 13;24(6):4221–39. doi: 10.1111/pbi.70623 (PMC13205892; doi:10.1111/pbi.70623)
Supplement: Supplementary file 1 — Figure S1: Chromatogram revealing the binding peptide of FvMAPK6, identified via pull‐down MS using recombinant FvMYB44.2‐GST. Figure S2: Generation of FvMAPK6 overexpression and gene‐edited strawberries. (A) FvMAPK6 expression levels in FvMAPK6‐OE lines (FvMAPK6‐OE‐2 and FvMAPK6‐OE‐3) using RT‐qPCR. (B) Kinase activity of FvMAPK6 in FvMAPK6‐OE red fruits, as detected using anti‐p44/42 antibody. (C) Diagram of the FvMAPK6 locus and target sites of the sgRNAs. The target sequences located at positions 25–44 bp, 97–116 bp and 366–384 bp within the coding sequence of FvMAPK6 were selected for CRISPR/Cas9‐mediated gene editing. Green boxes, exons; lines, introns. Two plants, biallelic Fvmapk6‐cr‐2 (with a 3‐bp deletion and a 5‐bp deletion for each allele) and Fvmapk6‐cr‐5 (homozygous for a 5‐bp deletion). In the T2 generation, a new biallelic plant (Fvmapk6‐cr‐2‐1) was identified with different edits compared to Fvmapk6‐cr‐2: one allele had a 5‐bp deletion while the other allele had a substitution of 1 bp along with a three‐bp deletion. (D) Kinase activity is decreased in Fvmapk6‐cr‐2 red fruits and completely abolished in Fvmapk6‐cr‐5 leaves. (E) Fvmapk6‐cr‐5 exhibits abnormal fertility and no fruit production. Scale bar, 6 cm. (F‐I) Validation of the biological function of FvMAPK6 in Fvmapk6‐cr‐2‐1 fruits by assessing fruit ripening (F), anthocyanin content (G), sugar content (H) and gene expression level (I) at 26 DAF. Scale bar, 0.6 cm. Values are means ± SD. in A (n = 3 biological replicates; each replicate contained ten leaves) and G, H, I (n = 3 biological replicates; each replicate contained 15 fruits). Statistical significance was determined by Student's t‐test (two‐sided, *p < 0.05, **p < 0.01, ns, no significance). Figure S3: Identification of the FvMAPKK4–MAPK6 phosphorylation cascade. (A) Yeast two‐hybrid (Y2H) assay to detect interactions between FvMAPK6 and FvMAPKKKs. The fusion vectors pGBKT7(BD)‐FvMAPKK1‐9 and pGADT7(AD) or pGADT7(AD)‐FvMAPK6 were co‐tr [file PBI-24-4221-s001.docx]

**Supporting Information**

Article title: FvMAPK6-Mediated FvMYB44s/FvSWEET1 Dual-Layer Regulation Modulates Sugar Accumulation in Strawberry Fruit, with *FvSPS3* Enabling Quality-Yield Balance

Authors: Qianqian Feng^a^, Lingzhi Wei^b^, Ting Liu^c^, Kexin Wang^a^, Xiaojing Li^a^, Chuang Liu^a^, Ronghui Sun^a^, Xia Li^a^, Zhaonan Yin^a^, Yanrong Wei^a^, Huazhao Yuan^d^, Qian Li^a^ and Bingbing Li^a,*^

The following Supporting Information is available for this article.

**Figure S1** Chromatogram revealing the binding peptide of FvMAPK6, identified via pull-down MS using recombinant FvMYB44.2-GST.

**Figure S2** Generation of *FvMAPK6* overexpression and gene-edited strawberries.

**Figure S3** Identification of the FvMAPKK4–MAPK6 phosphorylation cascade.

**Figure S4** Identification of the phosphorylated amino acids in FvMYB44.1 and FvMYB44.2 using LC-MS/MS.

**Figure S5** FvMYB44.1 directly regulates the expression of *FvCHS1*, *FvCHI*, *FvSWEET1*, *FvSPS3* and *FvSS2*.

**Figure S6** FvMAPK6 modulates the phosphorylation status of proteins involved in the SnRK1-TOR network as well as sugar transport, metabolism and signal transduction, as revealed by FvMAPK6-related phosphoproteomics.

**Figure S7** Identification of FvSWEET1 phosphorylation sites mediated by FvMAPK6 via phosphoproteomics.

**Figure S8** The expression pattern of *SWEET* gene in strawberry and the phylogenetic tree analysis of SWEET in strawberry, Arabidopsis thaliana and rice.

**Figure S9** Expression levels of *FvCHI*, *FvSPS3* and *FvSWEET1* in sucrose-treated fruits of WT and *FvMAPK6* transgenic lines determined by RT-qPCR.

**Figure S10** Generation and phenotypic analysis of *FvSPS3*-OE plants.

**Table S1.** Identification of specific FvMYB44.2-binding proteins by Pull down-MS (Partial Data).

**Table S2.** Selected differentially expressed genes (DEGs) in *Fvmapk6*-cr fruits compared to WT fruits.

**Table S3.** Identifying proteins involved in the accumulation of anthocyanin and sugar using IP-MS with anti-MAPK6 (Partial Data).

**Table S4.** Primers used in this study.


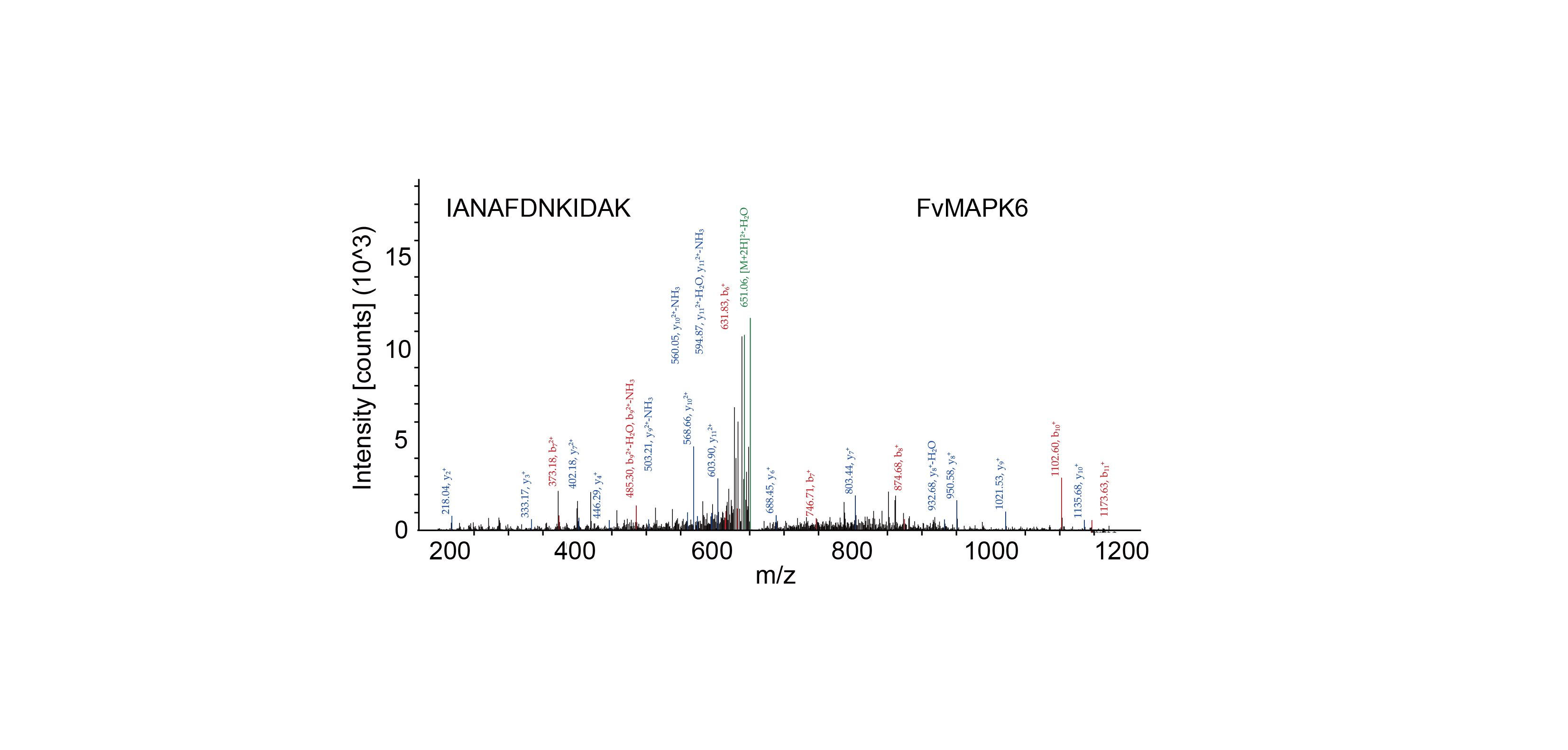


**Figure S1** **|** **Chromatogram revealing the binding peptide of FvMAPK6, identified via pull-down MS using recombinant FvMYB44.2-GST.**

**
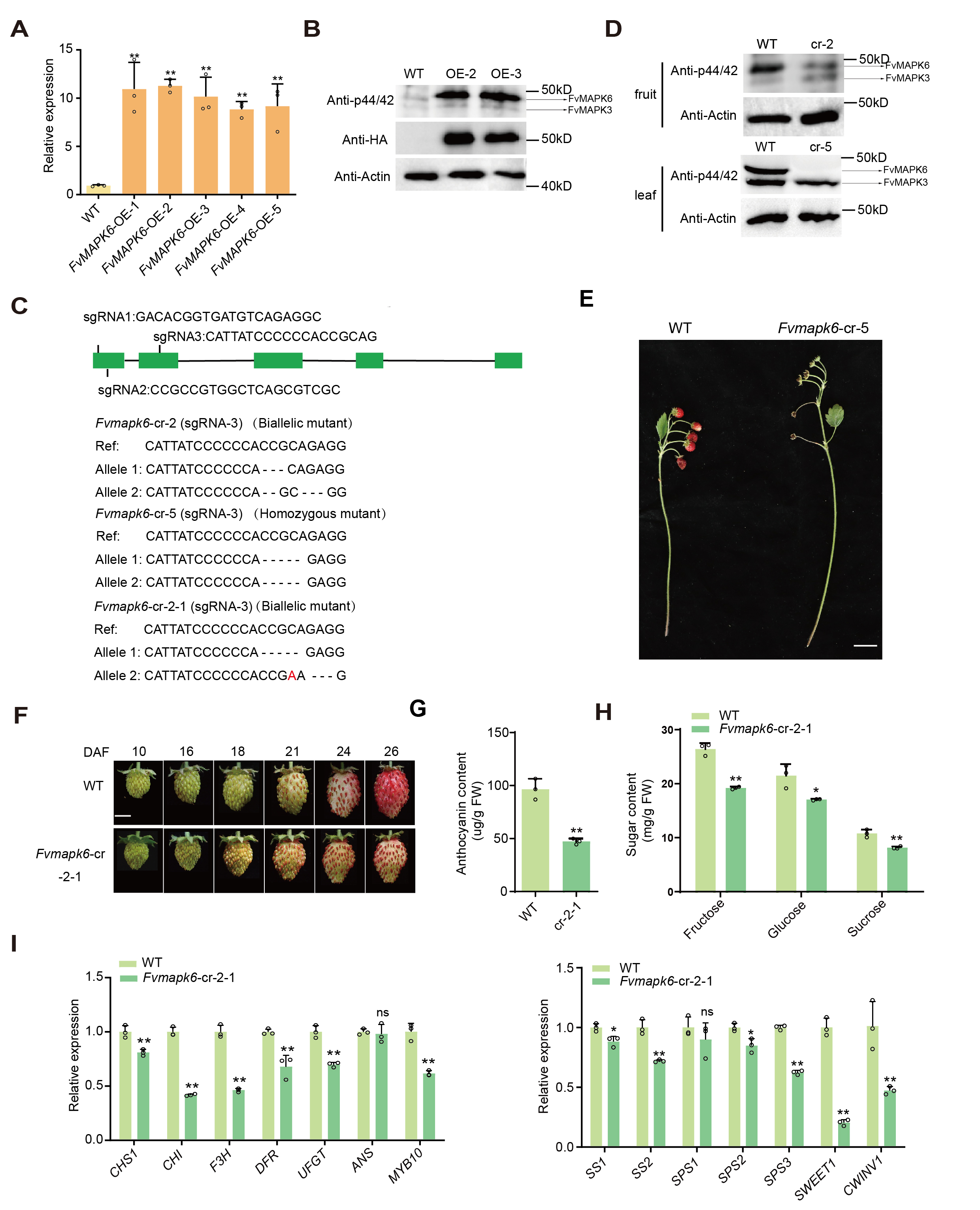
**

**Figure S2 | Generation of *FvMAPK6* overexpression and gene-edited strawberries.** **(A)** *FvMAPK6* expression levels in *FvMAPK6*-OE lines (*FvMAPK6*-OE-2 and *FvMAPK6*-OE-3) using RT-qPCR.

**(B)** Kinase activity of FvMAPK6 in *FvMAPK6*-OE red fruits, as detected using anti-p44/42 antibody.

**(C)** Diagram of the *FvMAPK6* locus and target sites of the sgRNAs. The target sequences located at positions 25-44 bp, 97-116 bp, and 366-384 bp within the coding sequence of *FvMAPK6* were selected for CRISPR/Cas9-mediated gene editing. Green boxes, exons; lines, introns. Two plants, biallelic *Fvmapk6*-cr-2 (with a 3-bp deletion and a 5-bp deletion for each allele) and *Fvmapk6*-cr-5 (homozygous for a 5-bp deletion). In the T2 generation, a new biallelic plant (*Fvmapk6*-cr-2-1) was identified with different edits compared to *Fvmapk6*-cr-2: one allele had a 5-bp deletion while the other allele had a substitution of 1 bp along with a three-bp deletion.

**(D)** Kinase activity is decreased in *Fvmapk6*-cr-2 red fruits and completely abolished in *Fvmapk6*-cr-5 leaves.

**(E)** *Fvmapk6*-cr-5 exhibits abnormal fertility and no fruit production. Scale bar, 6cm. **(F-I)** Validation of the biological function of FvMAPK6 in *Fvmapk6*-cr-2-1 fruits by assessing fruit ripening (F), anthocyanin content (G), sugar content (H), and gene expression level (I) at 26 DAF. Scale bar, 0.6 cm.

Values are means ± s.d. in A (*n*=3 biological replicates; each replicate contained ten leaves) and G, H, I (*n*=3 biological replicates; each replicate contained 15 fruits). Statistical significance was determined by Student’s t-test (two-sided, **P* < 0.05, ***P* < 0.01, ns, no significance).


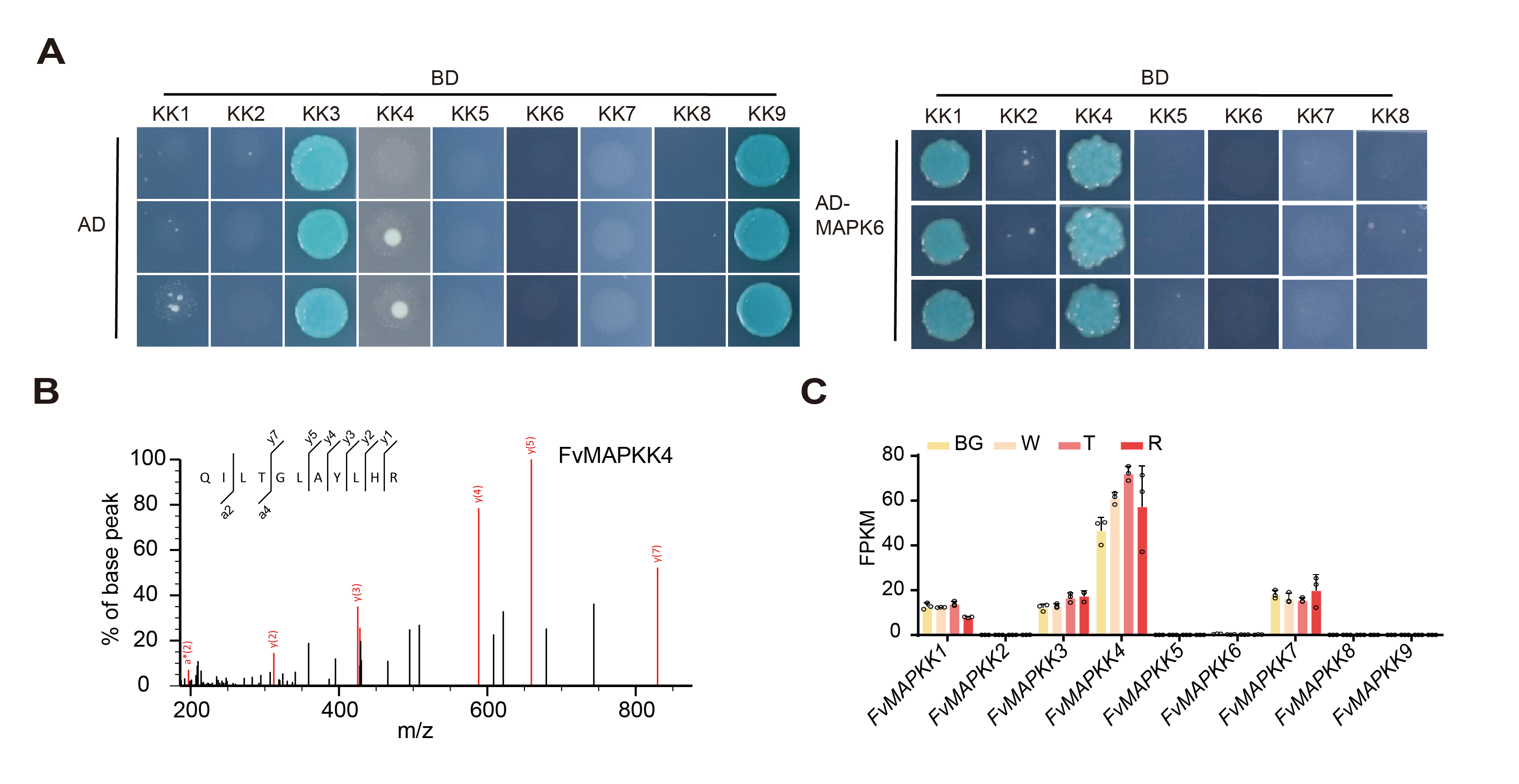


**Figure S3 |** Identification of the FvMAPKK4–MAPK6 phosphorylation cascade.

**(A)** Yeast two-hybrid (Y2H) assay to detect interactions between FvMAPK6 and FvMAPKKKs. The fusion vectors pGBKT7(BD)-FvMAPKK1-9 and pGADT7(AD) or pGADT7(AD)-FvMAPK6 were co-transformed into yeast cells, followed by growth on selection medium.

**(B)** Mass spectra of FvMAPKK4 peptides in *FvMAPK6*-OE fruits obtained by immunoprecipitation followed by mass spectrometry.

**(C)** Expression pattern of fruit-expressed *FvMAPKK*s in ‘Ruegen’ fruits at different developmental stages, as revealed by RNA-seq data. BG, big green fruit; W, white fruit; T, turning fruit; R, red fruit.


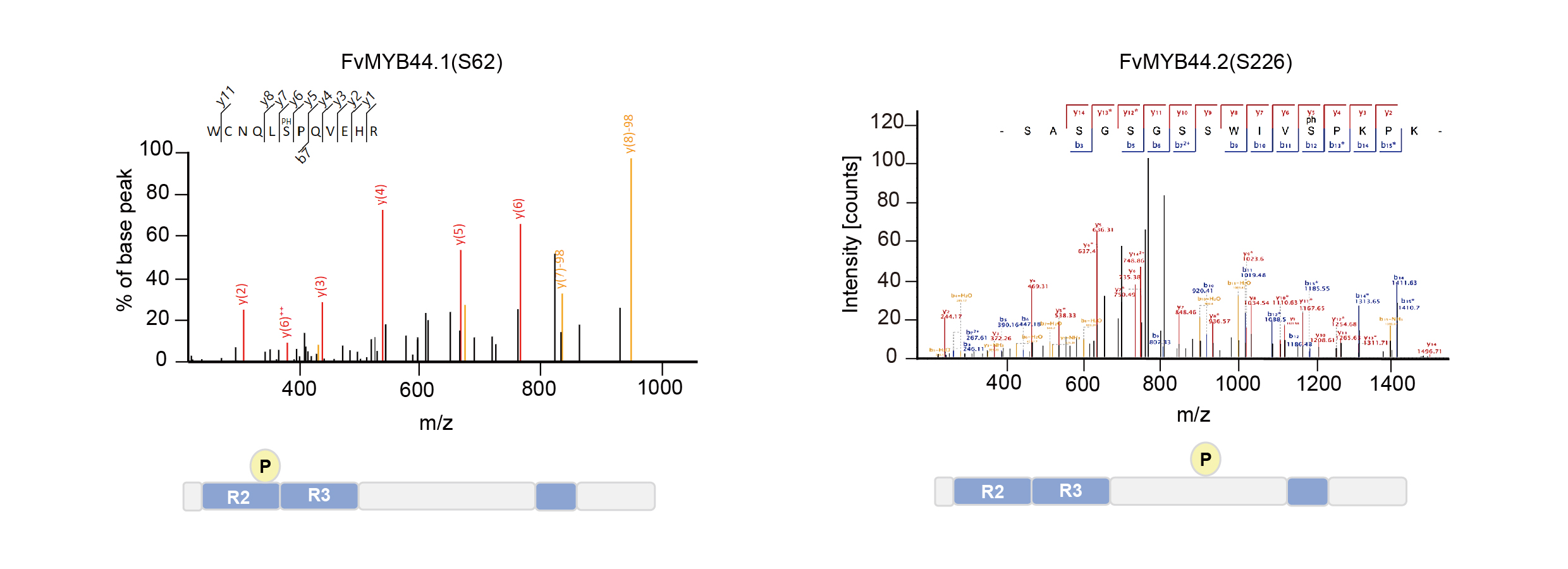


**Figure S4 | Identification of the phosphorylated amino acids in FvMYB44.1 and FvMYB44.2 using LC-MS/MS.**


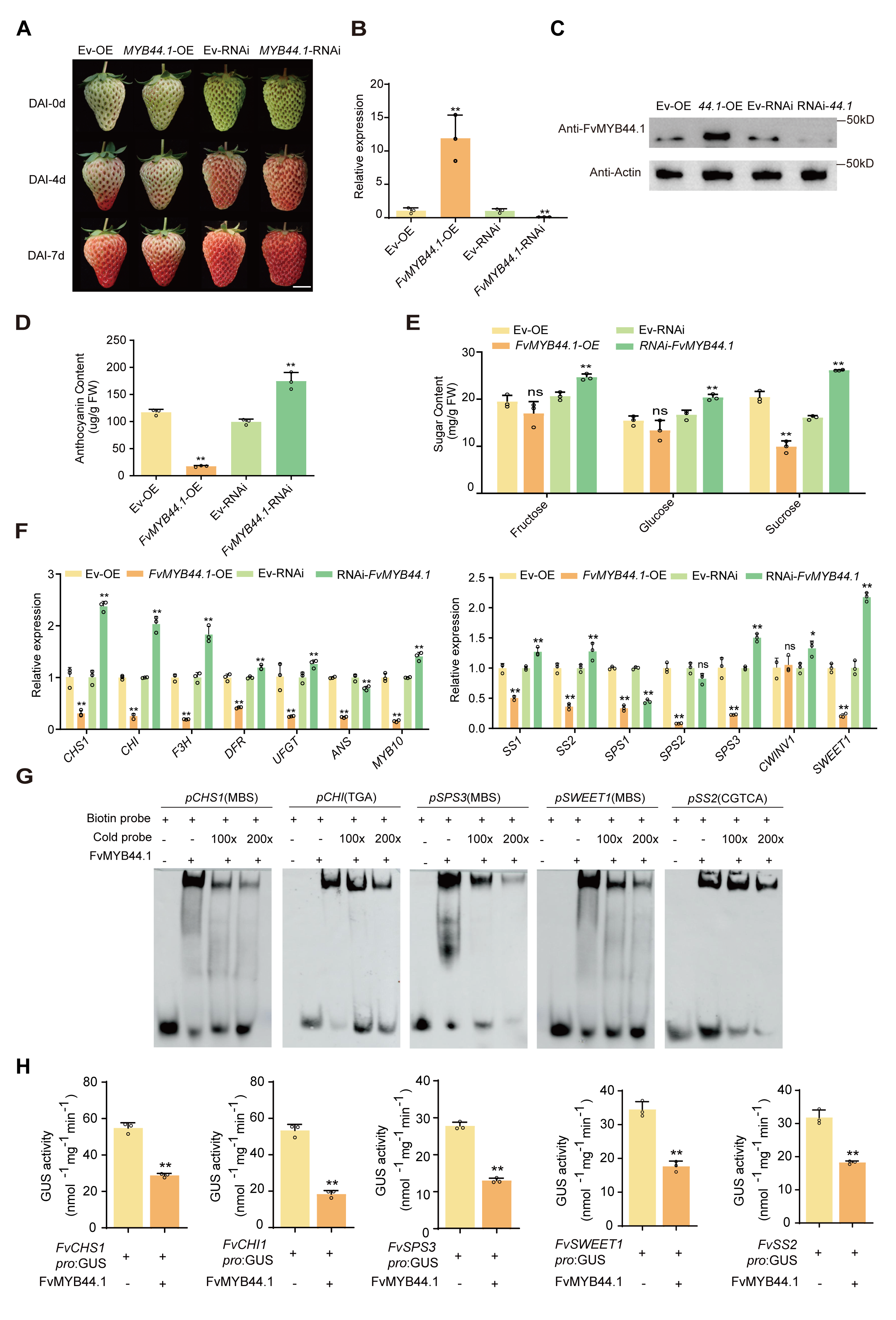


**Figure S5 | FvMYB44.1 directly regulates the expression of *FvCHS1*, *FvCHI*, *FvSWEET1*, *FvSPS3* and *FvSS2*.**

**(A)** Representative photographs of transiently overexpressing (*FvMYB44.1*-OE) and *FvMYB44.1*-silenced (*FvMYB44.1*-RNAi) ‘Benihoppe’ strawberry fruits. DAI, days after infiltration. Scale bar, 1 cm.

**(B, C)** RT-qPCR (B) and Western blot (C) analyses of FvMYB44.1 expression in *FvMYB44.1*-OE and *FvMYB44.1*-RNAi fruits.

**(D, E)** Anthocyanin (D) and sugar (E) contents in *FvMYB44.1*-OE and *FvMYB44.1*-RNAi fruits at 7 DAI.

**(F)** Relative expression levels of genes involved in anthocyanin biosynthesis and sugar metabolism in *FvMYB44.1*-OE and *FvMYB44.1*-RNAi fruits, are quantified by RT-qPCR; *FvACTIN* served as the internal reference gene.

**(G)** In vitro electrophoretic mobility shift assay (EMSA) demonstrating the binding affinity of recombinant FvMYB44.1-His to the promoters of *FvCHS1*, *FvCHI*, *FvSWEET1*, *FvSPS3*, and *FvSS2* following incubation of biotin-labeled probes and different concentrations of unlabeled probes followed by PAGE.

**(H)** GUS activity assay revealing that overexpressing *FvMYB44.1* suppresses of the expression of *FvCHS1,* *FvCHI*, *FvSWEET1*, *FvSPS3*, and *FvSUS2*. The *GUS* reporter gene was driven by each indicated promoter (*FvCHS1*, *FvCHI*, *FvSWEET1*, *FvSPS3*, *FvSUS2*). Transient co-infiltration experiments were conducted using 'Benihoppe' fruits with the *35S:FvMYB44.1* construct and the indicated *GUS* construct.

Values are means ± s.d. (*n*=3 independent biological replicates; each replicate contained 15 fruits). Significance was determined using Student’s t-test (two-sided, **P* < 0.05, ***P* < 0.01, ns, no significance).


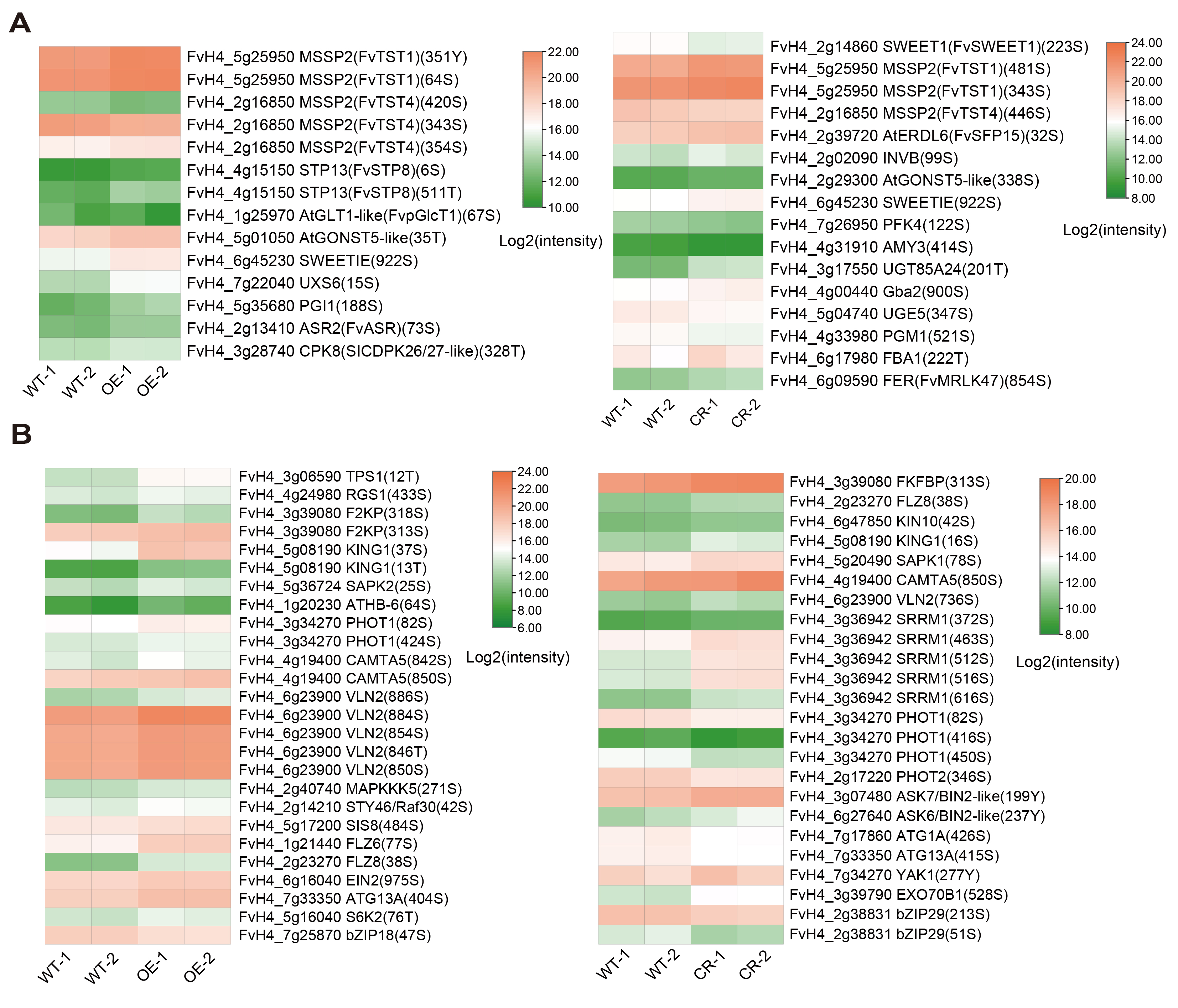


**Figure S6 | FvMAPK6 modulates the phosphorylation status of proteins involved in the SnRK1-TOR network as well as sugar transport, metabolism and signal transduction, as revealed by FvMAPK6-related phosphoproteomics. (A, B)** Heatmap representation of changes in phosphorylation of proteins associated with sugar transport, metabolism (A) and SnRK1-TOR network (B). The identification of related proteins in strawberries as presented in B, was based on the research progress made in other plant species. Specific members were determined through homology analysis (Huang et al. 2014; Cho et al. 2016; Shukla et al. 2018; Jamsheer et al. 2021; Van Leene et al. 2022; Gao, Zhang, et al. 2023; Liao et al. 2023).


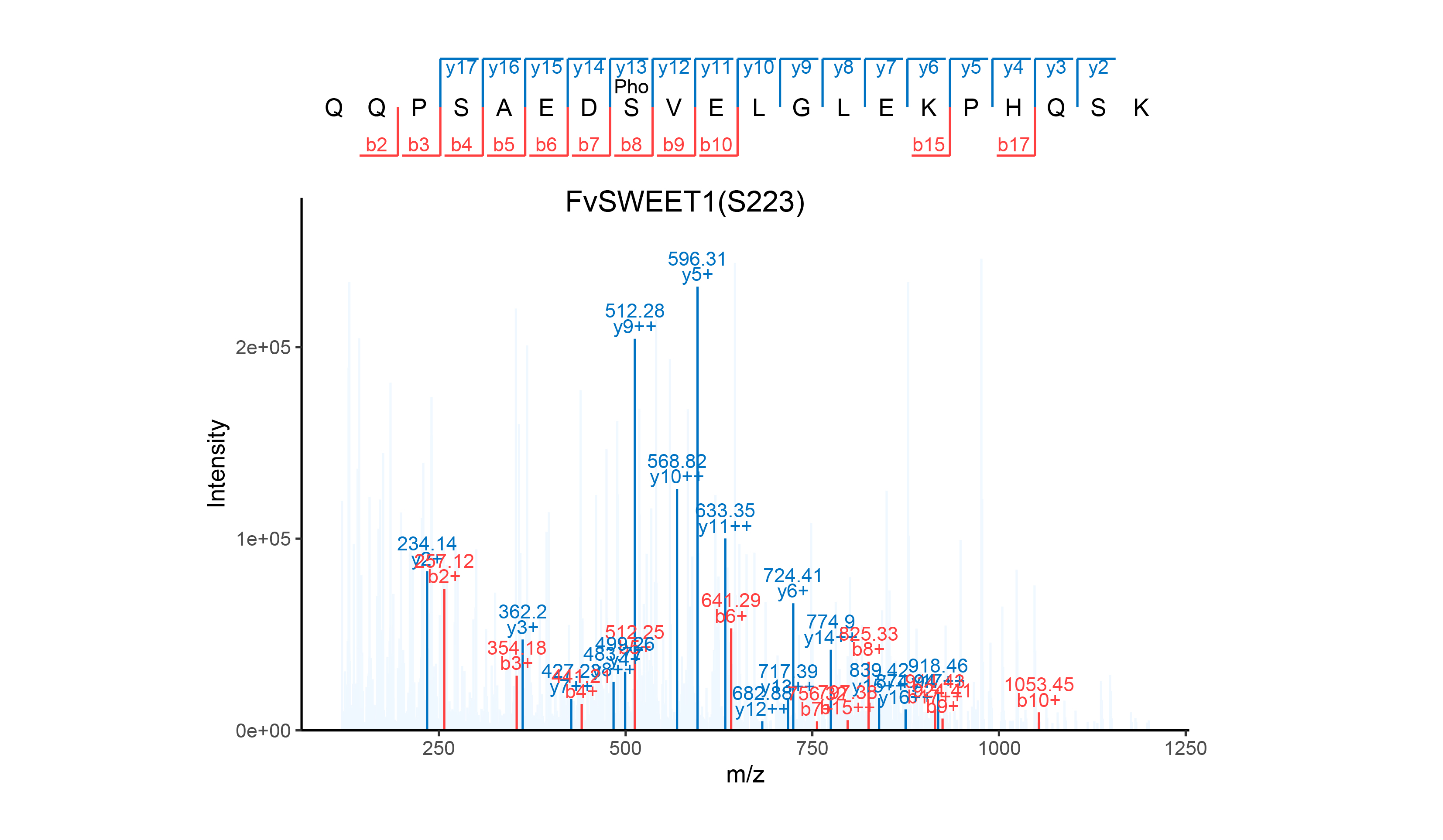


**Figure S7 | Identification of FvSWEET1 phosphorylation sites mediated by FvMAPK6 via phosphoproteomics.**

**
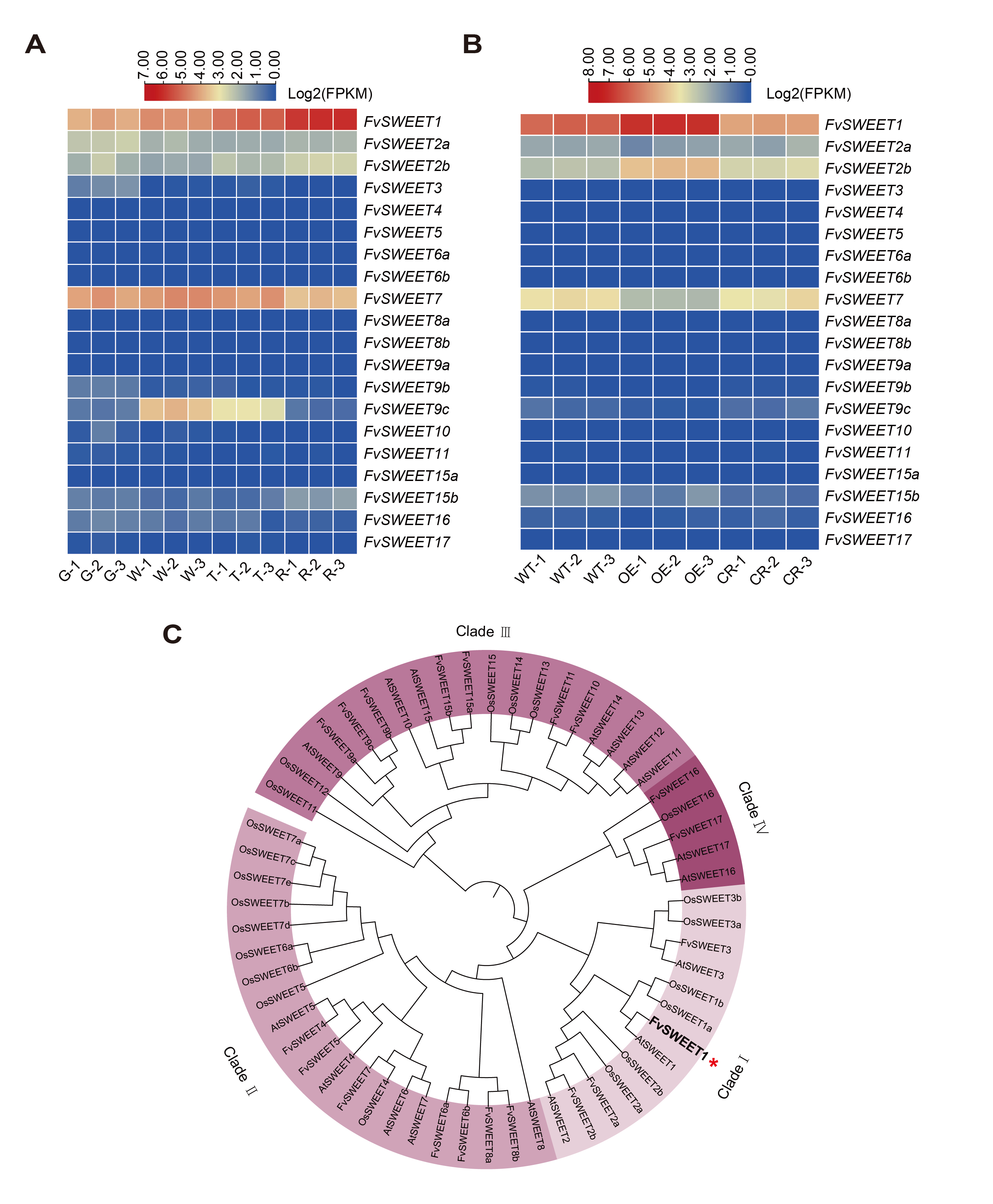
**

**Figure S8 | The expression pattern of *SWEET* gene in strawberry and the phylogenetic tree analysis of SWEET in strawberry, Arabidopsis thaliana and rice.**

**(A, B)** Heatmap representation of the expression levels for *FvSWEET*s based on RNA-seq data obtained from the fruits at different stages of development in ‘Ruegen’ strawberry (A) and the red fruits of WT, *FvMAPK6*-OE, and *Fvmapk6*-cr (B). G, green fruit; W, white fruit; T, turning fruit; R, red fruit; each number indicates a different replicate**.** The color scale represents the log_2_ FPKM values**.**

**(C)** Phylogenetic tree of FvSWEETs with Arabidopsis and rice orthologs reconstructed using the neighbor-joining method with MEGA version 11.0. FvSWEET1 is indicated by an asterisk (*).

**
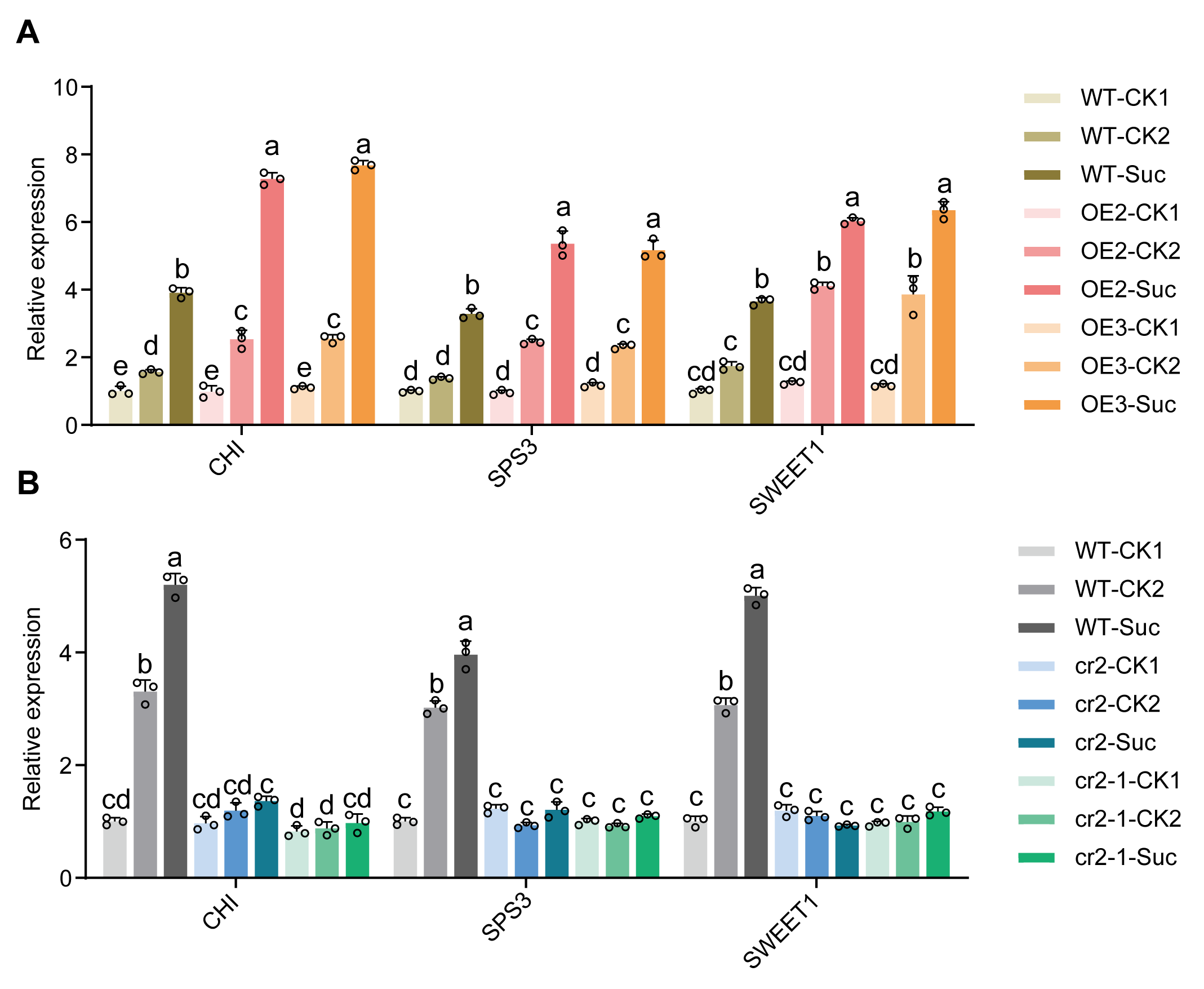
**

**Figure S9 | Expression levels of *FvCHI*, *FvSPS3* and *FvSWEET1* in sucrose-treated fruits of WT and *FvMAPK6* transgenic lines determined by RT-qPCR.** WT and *FvMAPK6*-OE fruits (A) or WT and *Fvmapk6*-cr fruits (B) were treated at the white fruit stage, with samples collected 2 days post-treatment. CK1, white fruit treated with water; CK2, white fruit treated with 50 mM mannitol; Suc, white fruit treated with 50 mM sucrose. Values are means ± s.d. (n=3 independent biological replicates; each replicate contained 10 fruits. Different lowercase letters indicate significant differences, as assessed by one-way ANOVA (with Tukey’s test) (*P* < 0.05).


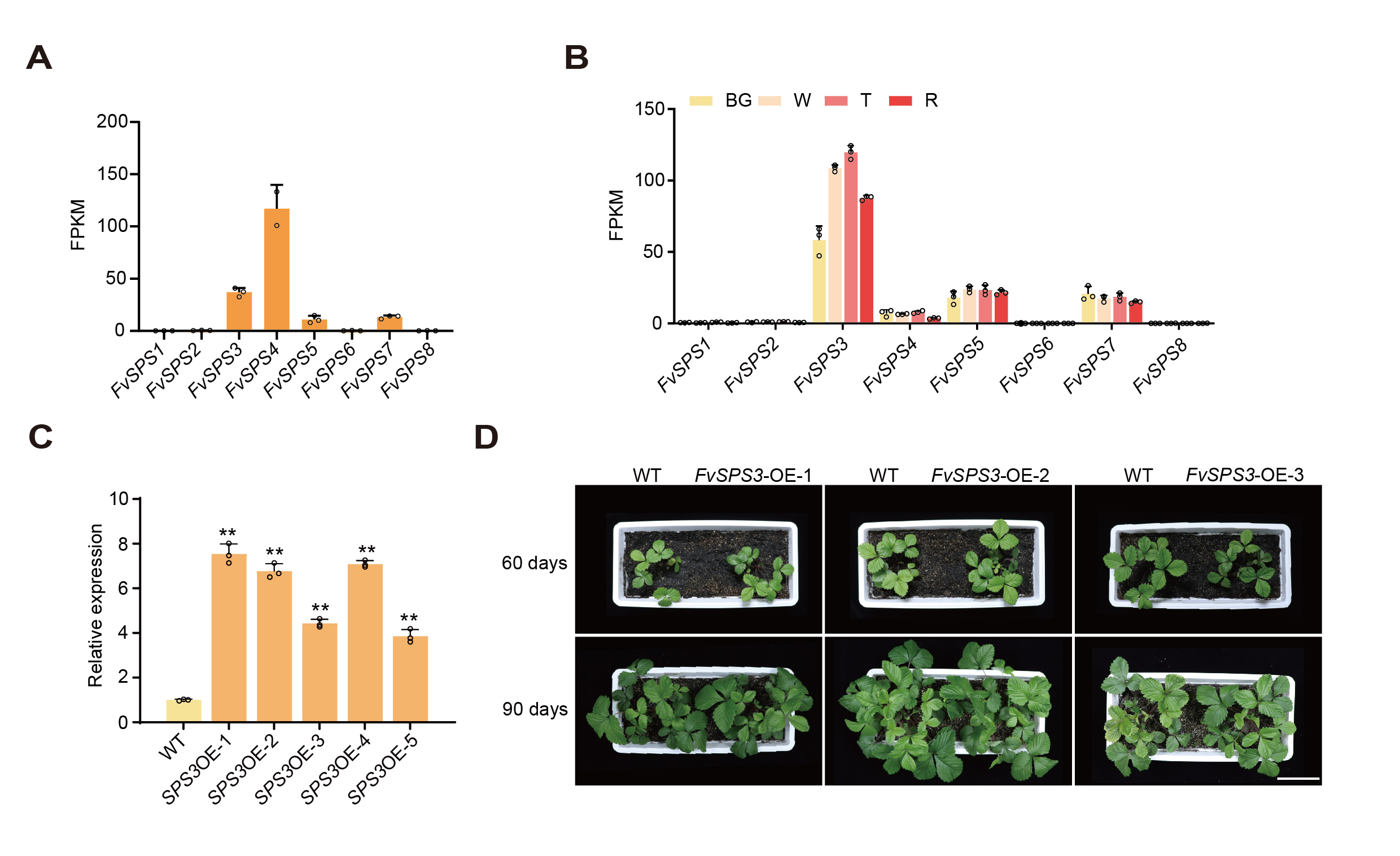


**Figure. S10 | Generation and phenotypic analysis of *FvSPS3*-OE plants.**

**(A, B)** *FvSPS* expression levels in ‘Ruegen’ leaves (A) and fruits (B), as determined by RNA-seq data. BG, big green fruit; W, white fruit; T, turning fruit; R, red fruit.

**(C)** *FvSPS3*-OE transgenic plants were identified by RT-qPCR.

**(D)** Representative photographs of WT and *FvSPS3*-OE plants. Scale bar, 8 cm.

Values are means ± s.d. (*n*=3 independent biological replicates; each replicate contained 10 leaves) in c. Significance was determined using Student’s t-test (two-sided, **P* < 0.05, ***P* < 0.01).

**Table S1.** **Identification of specific FvMYB44.2-binding proteins by Pull down-MS (Partial Data)**.

| **Accession** | **Peptides** | **Description** |
| --- | --- | --- |
| AHL46440.1 | 1 | PREDICTED:dihydroflavonol 4-reductase [Fragaria vesca] |
| XP_011470626.1 | 1 | PREDICTED: LRR receptor-like serine/threonine-protein kinase EFR [Fragaria vesca subsp. vesca] |
| **XP_004287157.1** | **1** | **PREDICTED: mitogen-activated protein kinase homolog D5 [Fragaria vesca subsp. vesca] (FvMAPK6)** |
| XP_004293398.1 | 1 | PREDICTED: SNF1-related protein kinase regulatory subunit beta-2 [Fragaria vesca subsp. vesca] |
| XP_004308141.1 | 1 | PREDICTED: subtilisin-like protease [Fragaria vesca subsp. vesca] |
| XP_011461248.1 | 1 | PREDICTED: UDP-glucuronic acid decarboxylase 5 isoform X2 [Fragaria vesca subsp. vesca] |
| XP_011470630.1 | 1 | PREDICTED: transcription factor MYB44-like [Fragaria vesca subsp. vesca] |
| XP_004307167.1 | 3 | PREDICTED: stromal 70 kDa heat shock-related protein, chloroplastic-like [Fragaria vesca subsp. vesca] |
| XP_004305069.1 | 1 | PREDICTED: signal recognition particle 54 kDa protein 2-like [Fragaria vesca subsp. vesca] |
| XP_004291618.1 | 1 | PREDICTED: protein SUPPRESSOR OF K(+) TRANSPORT GROWTH DEFECT 1 [Fragaria vesca subsp. vesca] |
| XP_011461810.1 | 1 | PREDICTED: protein STRICTOSIDINE SYNTHASE-LIKE 3-like, partial [Fragaria vesca subsp. vesca] |
| XP_011460234.1 | 1 | PREDICTED: nucleosome assembly protein 1;2 [Fragaria vesca subsp. vesca] |
| XP_004294578.1 | 1 | PREDICTED: histone H4-like [Fragaria vesca subsp. vesca] |
| XP_011460101.1 | 1 | PREDICTED: heterogeneous nuclear ribonucleoprotein 1-like isoform X2 [Fragaria vesca subsp. vesca] |
| XP_011467676.1 | 2 | PREDICTED: gamma aminobutyrate transaminase 3, chloroplastic isoform X2 [Fragaria vesca subsp. vesca] |
| XP_004289559.1 | 1 | PREDICTED: DNA polymerase alpha catalytic subunit [Fragaria vesca subsp. vesca] |

**Table S2. Selected differentially expressed genes (DEGs) in *Fvmapk6*-cr fruits compared to WT fruits.**

| **Functional category** | **Gene ID** | **Gene Name** | **OEvsWT Log2FC** | **CRvsWT Log2FC** |
| --- | --- | --- | --- | --- |
| Sugar biosynthesis and transporter | FvH4_1g09360 | *FvSUS1* | 2.36 | -0.09 |
|  | FvH4_2g26000 | *FvSUS2* | 1.65 | -0.10 |
|  | FvH4_3g03900 | *FvSTP4* | 2.58 | -0.36 |
|  | FvH4_4g15150 | *FvSTP8* | 1.79 | -0.66 |
|  | FvH4_1g01560 | *FvSTP10* | 1.63 | -0.43 |
|  | FvH4_2g24290 | *FvSFP11* | 2.68 | -0.35 |
|  | FvH4_2g08400 | *FvPMT1* | 1.59 | -0.91 |
|  | FvH4_3g00750 | *FvINT2* | 2.52 | -0.19 |
|  | FvH4_6g31830 | *FvpGlcT3* | -1.14 | -0.11 |
|  | FvH4_6g33590 | *FvCWINV1* | 3.52 | -1.66 |
|  | **FvH4_2g14860** | ***FvSWEET1*** | **1.00** | **-1.18** |
|  | FvH4_3g17630 | *FvSWEET2b* | 1.93 | 0.62 |
|  | FvH4_5g11560 | *FvSWEET7* | -1.47 | -0.08 |
|  | FvH4_2g24900 | *FvSWEET9c* | -1.80 | 0.36 |
| Flavonoid biosynthesis | FvH4_7g01160 | *FvCHS1* | 0.18 | -0.80 |
|  | FvH4_2g28040 | *FvCHS2* | 6.49 | -1.89 |
|  | FvH4_7g20870 | *FvCHI1* | 0.31 | -0.57 |
|  | FvH4_1g11810 | *FvF3H* | -0.31 | -0.96 |
|  | FvH4_2g39520 | *FvDFR* | 0.18 | -1.23 |
|  | FvH4_7g33840 | *FvUFGT* | -0.11 | -0.94 |
|  | FvH4_5g01170 | *FvANS1* | -0.07 | -1.28 |
| Transcription factors | FvH4_6g53770 | *FvWRKY50* | 3.31 | -1.46 |
|  | **FvH4_1g16770** | ***FvMYB44.1*** | **1.23** | **-0.21** |
|  | **FvH4_2g33810** | ***FvMYB44.2*** | **1.35** | **0.75** |
|  | **FvH4_2g05210** | ***FvMYB44.3*** | **2.71** | **0.79** |
|  | FvH4_5g17111 | *FvMYB1* | -1.28 | -0.75 |
|  | FvH4_7g32970 | *FvTTG1* | 2.44 | -0.11 |
|  | FvH4_2g13410 | *FvASR* | 0.70 | -1.02 |
|  | FvH4_6g53770 | *FvWRKY48* | 3.31 | -1.46 |

**Table S3. Identifying proteins involved in the accumulation of anthocyanin and sugar using IP-MS with anti-MAPK6 (Partial Data).**

| **Accession** | **OE-Score** | **WT-Score** | **Description** |
| --- | --- | --- | --- |
| XP_004307294.1 | 161 | #N/A | PREDICTED: UDP-glucose 6-dehydrogenase 1-like |
| XP_004290698.1 | 60 | #N/A | PREDICTED: sugar transporter ERD6-like 7 |
| XP_004296903.1 | 38 | #N/A | PREDICTED: sugar transport protein 13 |
| XP_004290813.1 | 81 | #N/A | PREDICTED: sucrose synthase 2 |
| XP_011463090.1 | 15 | #N/A | PREDICTED: FvSnRK1γ-like1; SNF1-related protein kinase regulatory subunit gamma-1-like |
| XP_004292060.1 | 22 | #N/A | PREDICTED: serine/threonine-protein kinase TOR |
| XP_004288068.1 | 36 | #N/A | PREDICTED: serine/threonine-protein kinase SRK2I |
| **XP_004303847.1** | **37** | **#N/A** | **PREDICTED: FvMAPKK4; mitogen-activated protein kinase kinase 5-like** |
| XP_004291365.1 | 43 | #N/A | PREDICTED: hexokinase-2, chloroplastic |
| XP_004287480.1 | 38 | #N/A | PREDICTED: hexokinase-1 |
| XP_004300322.1 | 77 | #N/A | PREDICTED: flavonol synthase 3-like |
| XP_004294328.1 | 29 | #N/A | PREDICTED: dihydroflavonol-4-reductase |
| XP_004297330.1 | 302 | 118 | PREDICTED: FvSnRK1γ-like2; SNF1-related protein kinase regulatory subunit gamma-1-like isoform X2 |
| XP_004287272.1 | 79 | 36 | PREDICTED: probable sucrose-phosphate synthase 1 |
| XP_004302537.1 | 49 | 28 | PREDICTED: FvMRLK47; receptor-like protein kinase FERONIA |
| XP_004303391.1 | 70 | 19 | PREDICTED: sucrose-phosphatase 1 |
| XP_004287157.1 | 89 | 15 | PREDICTED: FvMAPK6; mitogen-activated protein kinase homolog D5 |

**Table S4. Primers used in this study.**

| **Primer Name** | | **Forward (5’-3’)** | | **Reverse (5’-3’)** |
| --- | --- | --- | --- | --- |
| pSPYNE-FvMAPK6 | | GGAGAGAACACGGGGGACTCTAGAATGGAAGTCGGAGGTCAATCAGGAGA | | TGGGTACATCCCGGGAGCGGTACCATGCTGCTGATACTCAGGGTTAAA |
| pSPYCE-FvSWEET1 | | GGAGAGAACACGGGGGACTCTAGAATGGAAGACGTCGTCAAGTTCTTG | | TGGGTACATCCCGGGAGCGGTACCGACACCATTAACGCCGTTTGCTTG |
| pSPYCE-FvMYB44.1 | | GGAGAGAACACGGGGGACTCTAGAATGGCTATGAATCGGAAGGAAATGG | | TGGGTACATCCCGGGAGCGGTACCCTCAATTCTGCTGATCCCAATTCGC |
| pSPYCE-FvMYB44.2 | | GGAGAGAACACGGGGGACTCTAGAATGGCTTCTTCTTCAACCA | | TGGGTACATCCCGGGAGCGGTACCCTCCACCTTGCTAATCCCAAT |
| pCAMBIA1300-nLUC-M6 | | AACACGGGGGACGAGCTCGGTACCATGGAAGTCGGAGGTCAATCAGGAGA | | CCTTGTAGTCCATTTGTTGGATCCATGCTGCTGATACTCAGGGTTAAA |
| pCAMBIA1300-cLUC-44.1 | | TCGTACGCGTCCCGGGGCGGTACCATGGCTATGAATCGGAAGGAAATGG | | CCTTGTAGTCCATTTGTTGGATCCCTCAATTCTGCTGATCCCAATTCGC |
| pCAMBIA1300-cLUC-44.2 | | TCGTACGCGTCCCGGGGCGGTACCATGGCTTCTTCTTCAACCA | | CCTTGTAGTCCATTTGTTGGATCCCTCCACCTTGCTAATCCCAAT |
| pCAMBIA1300-cLUC-FvSWEET1 | | TCGTACGCGTCCCGGGGCGGTACCATGGAAGACGTCGTCAAGTTCTT | | CCTTGTAGTCCATTTGTTGGATCCGACACCATTAACGCCGTTTGCT |
| pCAMBIA1300-GFP-FvSWEET1 | | AATCGACTCTAGTCTAGAAAGCTTATGGAAGACGTCGTCAAGTTCTTG | | ATCCACTAGTATTTAAATGTCGACGACACCATTAACGCCGTTTGCTTG |
| pGEX-6P-1-FvMAPK6-F | | GGGCCCCTGGGATCCCCGGAATTCATGGAAGTCGGAGGTCAATCAGGAGA | | TCAGTCACGATGCGGCCGCTCGAGTCAATGCTGCTGATACTCAGGGTTAAA |
| pGEX-6P-1-FvMAPKK4-F | | GGGCCCCTGGGATCCCCGGAATTCATGAGGCCGAATAATCATATGAATC | | TCAGTCACGATGCGGCCGCTCGAGCTAAGACGGAAGCGGGCGCGGC |
| pET30a-FvMAPK6-F | | ATGGCTGATATCGGATCCGAATTCATGGAAGTCGGAGGTCAATCAGGAGA | | ATCTCAGTGGTGGTGGTGGTGGTGCTCGAGATGCTGCTGATACTCAGGGTTAAA |
| pET30a-FvMYB44.1-F | | ATGGCTGATATCGGATCCGAATTCATGGCTATGAATCGGAAGGAAATGG | | ATCTCAGTGGTGGTGGTGGTGGTGCTCGAGCTCAATTCTGCTGATCCCAATTCGC |
| pET30a-FvMYB44.2-F | | ATGGCTGATATCGGATCCGAATTCATGGCTTCTTCTTCAACCA | | ATCTCAGTGGTGGTGGTGGTGGTGCTCGAGCTCCACCTTGCTAATCCCAAT |
| pH7WG2D-FvMAPK6-HA | | | GGGGACAAGTTTGTACAAAAAAGCAGGCTGCATGGAAGTCGGAGGTCAATCAGGAGA | GGGGACCACTTTGTACAAGAAAGCTGGGTTCAAGCGTAGTCTGGGACGTCGTATGGGTAATGCTGCTGA |
| pH7WG2D-FvMYB44.1 | | | GGGGACAAGTTTGTACAAAAAAGCAGGCTGCATGGCTATGAATCGGAAGGAAATGG | GGGGACCACTTTGTACAAGAAAGCTGGGTTCACTCAATTCTGCTGATCCCAATTCGC |
| pH7WG2D-FvSPS3 | | | GGGGACAAGTTTGTACAAAAAAGCAGGCTGCATGCCGGAGGGTACCAGAACCG | GGGGACCACTTTGTACAAGAAAGCTGGGTTAGTTCTTGACAACCTCTAATTTCTCC |
| pFGC5941-FvMYB44.1-Ri-1 | | | CATTTACAATTACCATGGGGCGCGCCGACGACGGTAACGGTAACGGTGTA | GTAACATAAGAAATTCTTACACATTTAAATAGTCGACTCAACCACCGCCGCAA |
| pFGC5941-FvMYB44.1-Ri-2 | | | AGGACTCTAGGGACTAGTCCCGGGGACGACGGTAACGGTAACGGTGTA | ATTTGGATCCTAGGTGAGTCTAGAAGTCGACTCAACCACCGCCGCAA |
| CR-FvMAPK6-T1 | | | GACACGGTGATGTCAGAGGCGTTTTAGAGCTAGAAAT | GCCTCTGACATCACCGTGTCCAATCTCTTAGTCGACT |
| CR-FvMAPK6-T2 | | | CATTATCCCCCCACCGCAGGTTTTAGAGCTAGAAAT | CTGCGGTGGGGGGATAATGTGACCAATGTTGCTCC |
| CR-FvMAPK6-T3 | | | CCGCCGTGGCTCAGCGTCGCGTTTTAGAGCTAGAAAT | GCGACGCTGAGCCACGGCGGTGACCAATGGTGCTTTG |
| PDR196-SWEET1 | | | TCCCCCGGGCTGCAGGAATTCATGGAAGACGTCGTCAAGTTCTTG | GGGCCCCCCCTCGAGGTCGACTTAGACACCATTAACGCCGTTTGCTTG |
| pGADT7-FvMKK1 | | | GAATTCATGAACAAAGGAGGCTTGGGGTCTA | GGATCCTCAGCAGAGCTTTTGATTTTCCGGT |
| pGADT7-FvMKK2 | | | ATGGCCATGGAGGCCAGTATGTCGATGAGAAGCACTTCCGA | CTGCAGCTCGAGCTCGATTCAACCTCCATTGCCGGAGATAA |
| pGADT7-FvMKK3 | | | CATATGATGGCCGGACTAGAAGAACTG | CCCGGGTCACTGCAAAATATAAAGTTC |
| pGADT7-FvMKK4 | | | GAATTCATGAGGCCGAATAATCATATGAATC | GGATCCCTAAGACGGAAGCGGGCGCGGC |
| pGADT7-FvMKK5 | | | GAATTCATGGCTCTTCTCCAACGACGT | GGATCCTTAGTTTAGCCTAATACTTTTTGAG |
| pGADT7-FvMKK6 | | | GAATTCATGAAGACGAAGACGCCCTTGA | CCCGGGCTATCTGGGAAAATTTACAGG |
| pGADT7-FvMKK7 | | | ATGGCCATGGAGGCCAGTATGGCTCTCATCCGGGAACGC | CTGCAGCTCGAGCTCGATTCACTGTACGGCATCGGCTAGGGC |
| pGADT7-FvMKK8 | | ATGGCCATGGAGGCCAGTATGGCTCTTCTGCAACGACGTTGCAACA | | CTGCAGCTCGAGCTCGATCTAATGGTCTTCCCATGGGTTTGATCC |
| pGADT7-FvMKK9 | | GAATTCATGAGTACTGCAGCAGCTGACTTCAC | | CCCGGGTCAAATTATGAGCTCTTGATCAGTA |
| pGADT7-FvMAPK6 | | ATGGCCATGGAGGCCAGTGAATTCATGGAAGTCGGAGGTCAATCAG | | TCTACGATTCATCTGCAGCTCGAGTCAATGCTGCTGATACTCAGGG |
| pGBKT7-FvMKK1 | | CATATGGCCATGGAGGCCGAATTCATGAACAAAGGAGGCTTGGGGTCTA | | GTTATGCGGCCGCTGCAGGTCGACTCAGCAGAGCTTTTGATTTTCCGGT |
| pGBKT7-FvMKK2 | | CATATGGCCATGGAGGCCGAATTCATGTCGATGAGAAGCACTTCCGAGT | | GTTATGCGGCCGCTGCAGGTCGACACCTCCATTGCCGGAGATAAAT |
| pGBKT7-FvMKK3 | | CATATGGCCATGGAGGCCGAATTCATGGCCGGACTAGAAGAACTGAGGA | | GTTATGCGGCCGCTGCAGGTCGACTCACTGCAAAATATAAAGTTCTTG |
| pGBKT7-FvMKK4 | | CATATGGCCATGGAGGCCGAATTCATGAGGCCGAATAATCATATGAATC | | GTTATGCGGCCGCTGCAGGTCGACTAAGACGGAAGCGGGCGCGGCGG |
| pGBKT7-FvMKK5 | | CATATGGCCATGGAGGCCGAATTCATGGCTCTTCTCCAACGACGTCGCA | | GTTATGCGGCCGCTGCAGGTCGACTTAGTTTAGCCTAATACTTTTTGA |
| pGBKT7-FvMKK6 | | CATATGGCCATGGAGGCCGAATTCATGAAGACGAAGACGCCCTTGAATCT | | GTTATGCGGCCGCTGCAGGTCGACTCTGGGAAAATTTACAGGGGAT |
| pGBKT7-FvMKK7 | | CATATGGCCATGGAGGCCGAATTCATGGCTCTCATCCGGGAACGCCGCC | | GTTATGCGGCCGCTGCAGGTCGACTCACTGTACGGCATCGGCTAGGGC |
| pGBKT7-FvMKK8 | | CATATGGCCATGGAGGCCGAATTCATGGCTCTTCTGCAACGACGTTG | | GTTATGCGGCCGCTGCAGGTCGACCTAATGGTCTTCCCATGGGTTTGA |
| pGBKT7-FvMKK9 | | CATATGGCCATGGAGGCCGAATTCATGAGTACTGCAGCAGCTGACTTC | | GTTATGCGGCCGCTGCAGGTCGACTCAAATTATGAGCTCTTGATCAGT |
| Probe-PFaSPS3-MBS | | CTGACTAAACACGTAAAACAGTTGAAAACTAAAATGCAAT | | ATTGCATTTTAGTTTTCAACTGTTTTACGTGTTTAGTCAG |
| Probe-PCHS1-MBS | | AATTTAAATGACAACTGAAATTGGAATAAA | | TTTATTCCAATTTCAGTTGTCATTTAAATT |
| Probe-PSS2-CGTCA | | ATTTGGTCTAATGACGTCAGCCTCCCATCT | | AGATGGGAGGCTGACGTCATTAGACCAAAT |
| Probe-PSWEET-MBS | | CAATATTTACTGCAACAGTCTCCACTAGTC | | GACTAGTGGAGACTGTTGCAGTAAATATTG |
| Probe-PCHI-TGA | | TACAACCGGCCAAACGACACCATCTATTAC | | GTAATAGATGGTGTCGTTTGGCCGGTTGTA |
| 1301-HindIII-PFaSPS3-F | | GTCGACCTGCAGGCATGCAAGCTTAGCGTCTACTCTCCACAGTTTCCTGCC | | AAATTTACCCTCAGATCTACCATGGCGTCGGAGCTCGAATTCAGACCAAATC |
| 1301-HindIII-PSWEET1-F | | GTCGACCTGCAGGCATGCAAGCTTTAGGATTAAAAACTCCTGATATTA | | AATTTACCCTCAGATCTACCATGGATATGGAGGCTCTGATGCGGAAAG |
| 1301-HindIII-PSS2-F | | GTCGACCTGCAGGCATGCAAGCTTAGCACACGATCCATGTCCACTAAT | | AATTTACCCTCAGATCTACCATGGGGTTGAATTGCAATTGCAAGCTTG |
| 1301-HindIII-PCHS1-F | | GTCGACCTGCAGGCATGCAAGCTTTTATGCTGATTTGATTATGTGTTT | | AAATTTACCCTCAGATCTACCATGGTTTGATTTCTCAGAGAAGTGTCGA |
| 1301-HindIII-PCHI-F | | GTCGACCTGCAGGCATGCAAGCTTTGCAGTGAGAGATTGGTCTGATTT | | AAATTTACCCTCAGATCTACCATGGTTGATTTTCTTGGTTTTGATGTAA |
| pBI121-FvMYB44.1 | | TCTAGAATGGCTATGAATCGGAAGGAAATGG | | GGATCCTCACTCAATTCTGCTGATCCCAATTCGC |
| pBI121-FvMYB44.2 | | TCTAGAATGGCTTCTTCTTCAACCA | | GGATCCTCACTCCACCTTGCTAATCCCAAT |
| pBI121-FvMAPK6 | | TCTAGAATGGAAGTCGGAGGTCAATCAGGAGA | | GGATCCTCAATGCTGCTGATACTCAGGGTTAAA |
| 1301-35S-FvMYB44.1 | | TTCGAGCTCGGTACCCGGGGATCCTGAGACTTTTCAACAAAGGGTAAT | | AAGCTTGCATGCCTGCAGGTCGACTCACTCAATTCTGCTGATCCCAAT |
| 1301-35S-FvMYB44.2 | | TTCGAGCTCGGTACCCGGGGATCCTGAGACTTTTCAACAAAGGGTAAT | | AAGCTTGCATGCCTGCAGGTCGACCTACTCCACCTTGCTAATCCCAAT |
| 1301-35S-FvMAPK6-R | | TTCGAGCTCGGTACCCGGGGATCCTGAGACTTTTCAACAAAGGGTAAT | | AAGCTTGCATGCCTGCAGGTCGACTCAATGCTGCTGATACTCAGGGTTAAA |
| qRT-FvACTIN | | GCCAACCGTGAGAAGATG | | TCCAGAGTCAAGAACAATACCAG |
| qRT-FvMPAK6 | | TCCCTACTTGACATCTCTCCAC | | CATTTGTTCCTCAGACAGTGC |
| qRT-FvCHS1 | | CATACCCCGACTACTACTTTCGT | | CGCACATACTGGGATTCTCTT |
| qRT-FvCHI | | AGCGAAAGCCATTGAAAAGT | | CATTTGGTGATTGTGTGAAGAG |
| qRT-FvDFR | | ACCCTGAGAACGAAGTGATAAAG | | TAAACACCACCCTCCGAACT |
| qRT-FvF3H | | CTTTCGTGGTGAATCTTGGAG | | TCGCTATGGACAACCTGCT |
| qRT-FvUFGT | TAGAGGATGTGTGGAAGATTGGT | | | CTGTTGTGCGAGTTGTTTTAGTG |
| qRT-FvANS | CTTGGCTTGGGATTAGAAGAAG | | | TGAGGGCATTTTGGGTAGTAGT |
| qRT-FvMYB10 | CAACAGCACCACCACAGACT | | | GCTTGCCGATTGTACCGTAT |
| qRT-FvSUS1 | CCCTGATTCTGACCTTTACTGG | | | GATGATGAAGTCGGTGTGGTT |
| qRT-FvSUS2 | TTGCTGAGGATGTTGCAGGT | | | AACAACGAGGCGACGAGATT |
| qRT-FvSPS1 | GAGCCTTGAATGTCCCAATGT | | | ATCTCCTGTCTGGTGCTGGTT |
| qRT-FvSPS2 | CAAAGAAGTACTCTGGCCCCAT | | | GCCTTTGCCAATTTGTTCACC |
| qRT-FvSPS3 | CGTAGATTGGAGTTATGGAG | | | CGAATGATGTAAGAACCACTGC |
| qRT-FvSWEET1 | GGTAATGCCACCGCTCTCTT | | | CACCAGAATGTTGTTCGGCG |
| qRT-FvSWEET2b | TTTGGGCTGTTTGTTTCGCC | | | TTGAAACACTGCACCGACTG |
| qRT-FvSWEET7 | CTGCATACGCTCTCATCCGT | | | TGACAAGTTCACCTCCCTGC |
| qRT-FvSWEET9c | CGCCATGTTGACGCTCTACT | | | ACATCGTTTGGGATGAGGCA |
| qRT-FvCWINV1 | GGGTCCGTCACAATCCTTCC | | | TCCCTAAGAAACGGGTCGGA |
